# Activation of the integrated stress response and loss of cFLIP_L_ under glutamine limitation induce IL-8 gene expression and secretion in glutamine-dependent tumor cells

**DOI:** 10.1038/s41420-025-02625-3

**Published:** 2025-07-19

**Authors:** Rocío Mora-Molina, F. Javier Fernández-Farrán, Abelardo López-Rivas, Carmen Palacios

**Affiliations:** 1 Centro Andaluz de Biología Molecular y Medicina Regenerativa-CABIMER, CSIC-Universidad de Sevilla-Universidad Pablo de Olavide, Sevilla, Spain; 2 Present Address: Epigenetics, Babraham Institute, Cambridge, UK

**Keywords:** Cancer metabolism, Cancer microenvironment

## Abstract

Growing evidence suggests that the proapoptotic TNF-related apoptosis-inducing ligand receptor 2 (TRAIL-R2/DR5) signaling pathway can also trigger the production of inflammatory cytokines, thereby promoting tumor progression. We recently reported that glutamine depletion impacts the survival of glutamine-dependent tumor cells by activating the TRAIL-R2/DR5-mediated apoptotic machinery. However, it remains unclear whether glutamine limitation activates a TRAIL-R2/DR5-regulated inflammatory response. In this study, we demonstrate that glutamine starvation activates two parallel signaling pathways, leading to the gene expression and secretion of the pro-angiogenic and pro-inflammatory interleukin-8 (IL-8/CXCL8) in tumor cells. Our findings reveal that the amino acid-sensing general control nonderepressible-2 kinase (GCN2)/activating transcription factor 4 (ATF4) signaling axis contributes to the upregulation of IL-8 gene expression in glutamine-deprived tumor cells. Furthermore, our results indicate that the loss of the long isoform of cellular FLICE-inhibitory protein (cFLIP_L_), which occurs as result of the metabolic stress induced by glutamine limitation, promotes TRAIL-independent activation of the NF-kB pathway via TRAIL-R2/DR5, a key mechanism driving the observed IL-8 upregulation under starvation conditions. Given the severe depletion of glutamine observed in growing tumors, our data suggest that IL-8 secretion, induced by this metabolic stress, may play a significant role in activating inflammatory and angiogenic responses, thereby counteracting apoptosis and ultimately promoting tumor progression.

## Introduction

The growth and survival of cancer cells depend on metabolites and metabolic pathways that differ from those of healthy cells. A hallmark of many cancers is the increased uptake and utilization of glutamine, which is crucial for governing redox homeostasis, supporting energy production, and driving rapid growth [1]. In the context of a tumor, the combination of irregular blood vessel formation and high glutamine consumption by cancer cells leads to selective depletion of this amino acid in the tumor microenvironment [2]. Additionally, a recent metabolomic study revealed that glutamine levels are significantly lower in tumor tissues than in adjacent benign tissues [3]. Furthermore, the central region of solid tumors is deficient in glutamine relative to other amino acids [4].

In response to amino acid deprivation, a common challenge encountered by rapidly proliferating tumor cells, these cells can activate the integrated stress response (ISR) to address stress and restore homeostasis, thereby ensuring cell viability and proliferation [5, 6]. The initial step in this signaling pathway is the phosphorylation of eukaryotic translation initiation factor 2 alpha (eIF2α) by the GCN2 kinase [7, 8], which inhibits cap-dependent global protein synthesis while permitting the selective translation of key downstream factors that orchestrate an adaptive gene expression program [9]. This program is essential for the survival and proliferation of tumor cells under conditions of nutrient deprivation [10–12]. However, under prolonged starvation, GCN2 can trigger the ISR to induce apoptosis, thereby eliminating chronically stressed cells through the ATF4/CHOP axis [13–16]. In certain scenarios, GCN2 can also influence immune responses, particularly in the context of inflammation [17], although the specific signaling mechanisms involved remain poorly understood.

Tumor necrosis factor-related apoptosis-inducing ligand (TRAIL), a member of the tumor necrosis factor (TNF) family [18], can specifically induce apoptosis in a variety of cancer cells by binding to pro-apoptotic receptors [19, 20]. Upon activation of TRAIL receptors by TRAIL, a death-inducing signaling complex (DISC) is formed, which is crucial for the activation of caspase-8 and the initiation of apoptosis [21]. The cFLIP proteins can also be recruited to this complex, where they inhibit the processing and activation of caspase-8 [22]. However, a cell-type-specific pro-apoptotic role has also been described for the long isoform, which is dependent on the ratio of caspase-8 to c-FLIP_L_ [23]. Thus, the levels of this isoform serve as crucial regulators of DISC output [23]. Interestingly, several studies have shown that TRAIL receptors can also trigger the production of inflammatory cytokines under certain conditions [24–28]. Upon binding of TRAIL to death receptors, a secondary cytoplasmic complex called the FADDosome may form to activate the NF-κB-dependent production of inflammatory cytokines by promoting the ubiquitination of RIPK1 [27]. This proinflammatory function is largely dependent on the scaffolding role of caspase-8 and is further amplified when caspase-8 activity is inhibited [26, 27]. Recently, cFLIP_L_ was shown to inhibit NF-κB activation and the production of inflammatory cytokines induced by Fas or TRAIL by blocking the formation of FADDosome complexes [24]. Thus, cFLIP_L_ may function as an inhibitor of both apoptosis and inflammation through distinct mechanisms.

Interleukin-8 (IL-8) is a chemokine originally identified as a neutrophil chemoattractant during acute inflammation [29]. Beyond this role, IL-8 also regulates angiogenesis by promoting endothelial cell proliferation, survival, and migration, ultimately leading to the formation of new blood vessels [30]. It is produced in response to infection or tissue injury [31] but can also be induced by death ligands, such as tumor necrosis factor alpha (TNFα), Fas/CD95 ligand, and TRAIL [24, 26, 27]. Additionally, IL-8 can be released by cells undergoing various forms of stress, including endoplasmic reticulum (ER) stress [28] and metabolic stress [32, 33]. Notably, IL-8 has been shown to exert stimulatory effects on cancer stem cells in breast [34], colon [35], and pancreatic [36] cancers via its receptors CXCR1/2. Given these findings, IL-8 is considered to play a significant role in tumor progression.

In this study, we explored the potential regulation of IL-8 gene expression and secretion in tumor cells deprived of glutamine, as well as the signaling pathways involved. Our results reveal that glutamine limitation triggers a GCN2-ATF4 signaling cascade that contributes to IL-8 production, working in concert with the activation of the NF-kB pathway by a caspase-8-dependent mechanism. Importantly, our findings highlight that TRAIL-R2/DR5, regardless of its ligand, along with the downregulation of cFLIP_L_, plays a critical role in activating NF-κB and subsequently promoting IL-8 secretion under glutamine starvation conditions.

## Results

### Glutamine deprivation induces proinflammatory IL-8 gene expression and secretion through both GCN2 and TRAIL-R2/DR5-activated signaling pathways

**Fig. 1:**
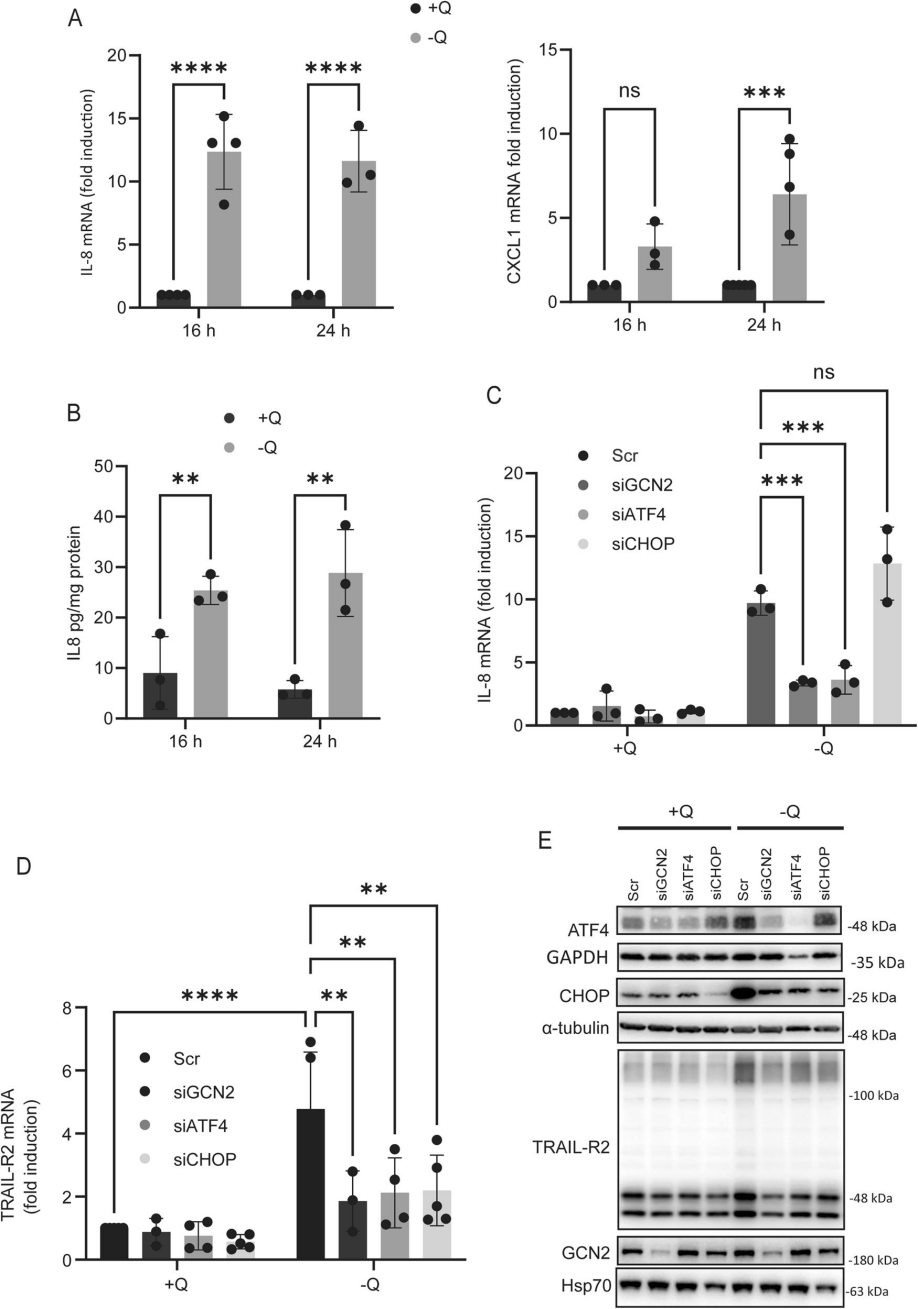
Glutamine deprivation induces IL-8 synthesis and secretion through the GCN2-ATF4 pathway. **A** HCT116 cells were cultured in the presence or absence of glutamine for 16 and 24 h and IL-8 and CXCL1 mRNA levels were assessed via RT‒qPCR as described in the Materials and Methods section. Relative mRNA levels were determined by comparison to those observed under glutamine-supplemented conditions at each corresponding time point. **B** HCT116 cells were treated as described in **A**) and the resulting supernatants were analysed for IL-8 production by ELISA as described in the Materials and Methods. In **C**-**E** HCT116 cells were transfected with either a scrambled oligonucleotide or siRNAs targeting GCN2, ATF4 or CHOP. Forty-eight hours after transfection, the cells were incubated for 16 h in complete or glutamine-deprived medium, and the IL-8 (**C**) or TRAIL-R2/DR5 (**D**) mRNA levels were assessed by RT‒qPCR. Relative levels of IL-8 or TRAIL-R2/DR5 mRNA were quantified relative to those in scrambled siRNA-transfected cells under glutamine-supplemented conditions. ATF4, CHOP, TRAIL-R2/DR5 and GCN2 protein levels were assessed by western blotting after 16 h of treatment (**E**). α-Tubulin, Hsp70 and GAPDH were used as protein loading controls. Data are presented as mean ± SD from at least three independent experiments and were analyzed using two-way ANOVA. **P < 0.01; ***P < 0.001; ****P < 0.0001; ns not statistically significant.

In addition to its role in apoptosis signaling, TRAIL-R2/DR5 activation can also stimulate the synthesis and/or release of proinflammatory cytokines in various tumor or transformed cell lines [24–28]. In a previous work, we demonstrated that glutamine deprivation induces cell death in glutamine-addicted tumor cell lines through the activation of a GCN2-stimulated, TRAIL-R2/DR5-mediated apoptotic signaling pathway [16]. Here, we investigated the production of proinflammatory cytokines in response to glutamine deprivation in HCT116 cells, a glutamine-addicted human colon cancer cell line [37]. Removal of glutamine from the culture medium led to the upregulation of IL-8 gene expression (Fig. 1A, left panel) and increased IL-8 secretion (Fig. 1B) in HCT116 cells. While some induction of CXCL1 mRNA was observed under starvation conditions (Fig. 1A, right panel), CXCL1 protein was undetectable in the supernatants of cells cultured without glutamine. IL-6 mRNA levels in HCT116 cells, whether cultured with or without glutamine, consistently remained below the detection threshold, as determined by qPCR (amplification cycles ≥30).

GCN2 is a stress-responsive kinase that becomes activated by uncharged tRNAs [38] during amino acid deprivation. This signaling pathway primarily aims to restore cellular homeostasis by inhibiting general cap-dependent protein translation while selectively enhancing the translation of specific mRNAs, such as the transcription factor ATF4, to promote cellular recovery. However, under prolonged or severe stress conditions, cell death can also be triggered by the activation of CHOP (GADD153) gene expression, a downstream target of ATF4 [16, 39]. To investigate the role of the GCN2-ATF4-CHOP pathway in the proinflammatory response triggered by glutamine deprivation in HCT116 cells, we examined the impact of knocking down the expression of these three proteins on IL-8 gene expression. As shown in Fig. 1C, knockdown of GCN2 or ATF4 significantly inhibited IL-8 upregulation in response to glutamine depletion. In contrast, CHOP silencing had no effect on IL-8 induction in glutamine-deprived HCT116 cells. Downstream of CHOP, the proapoptotic receptor TRAIL-R2/DR5 is known to be upregulated in response to various cellular stressors [16, 40–43]. TRAIL-R2/DR5 expression was significantly increased at both the mRNA (Fig. 1D) and protein (Fig. 1E) levels upon glutamine deprivation. However, this upregulation was abolished in cells deficient for GCN2, ATF4, or CHOP, indicating that TRAIL-R2/DR5 expression in HCT116 cells is regulated through this pathway. Collectively, our findings demonstrate that while CHOP-mediated TRAIL-R2/DR5 upregulation occurs in response to glutamine starvation, it is not required for IL-8 induction under these conditions.

**Fig. 2:**
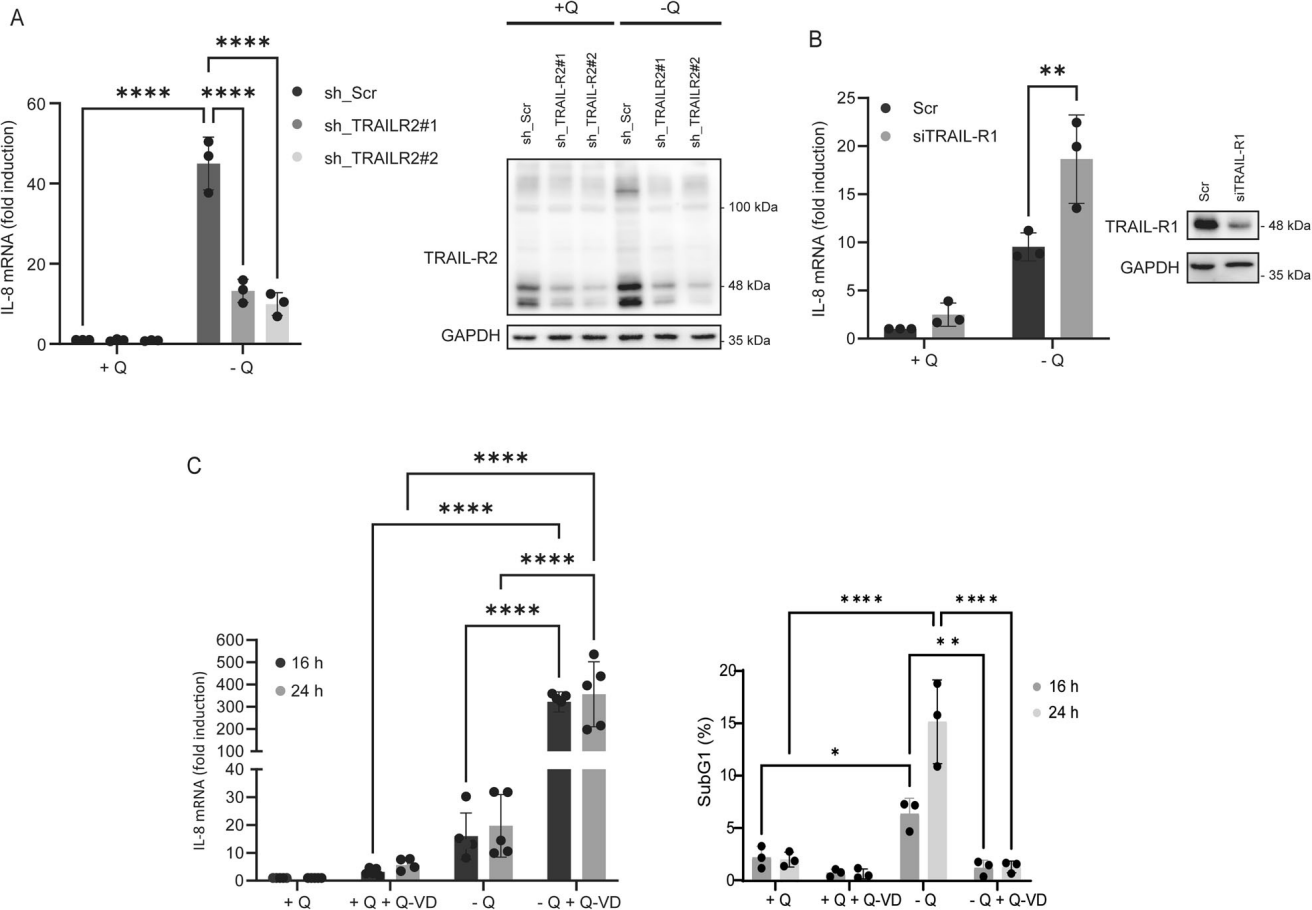
TRAIL-R2/DR5 is required for IL-8 induction under glutamine-deprived conditions, whereas caspase activity is not essential. **A** HCT116 cells stably expressing shScrambled, shTRAIL-R2#1, or shTRAIL-R2#2 were exposed to either glutamine-replete or glutamine-deprived conditions, as shown in the graph. IL-8 mRNA levels were quantified by RT-qPCR following 24 hours of treatment. Relative IL-8 mRNA levels were determined in comparison to those in shScrambled cells cultured under glutamine-replete conditions (left panel). TRAIL-R2/DR5 knockdown was confirmed in whole-cell extracts by western blotting (right panel). GAPDH levels were used as protein loading controls. **B** HCT116 cells were transfected with either a scrambled oligonucleotide (Scr) or siRNA targeting TRAIL-R1/DR4. Forty-eight hours after transfection, the cells were incubated for 24 h in complete or glutamine-deprived medium, and the IL-8 mRNA levels were assessed by RT‒qPCR. Relative IL-8 mRNA levels were determined in comparison to those in scrambled cells cultured under glutamine-replete conditions. **C** HCT116 cells were cultured under glutamine-replete or glutamine-deprived conditions, in the presence or absence of 20 µM Q-VD, as indicated in the graphs. IL-8 mRNA levels (left panel) were assessed by RT-qPCR, and relative IL-8 mRNA levels were determined in comparison to the glutamine-containing condition at each time point. Apoptosis (right panel) was assessed by subG1 analysis, as described in the Materials and Methods section, after 16 or 24 h of treatment, as indicated. Data are presented as mean ± SD from at least three independent experiments and were analyzed by two-way ANOVA. *P < 0.05; **P < 0.01; ****P < 0.0001.

TRAIL-R2/DR5 plays a pivotal role in apoptosis induced by glutamine deprivation in various cancer cell lines [16]. Although TRAIL-R2/DR5 upregulation did not appear to contribute to IL-8 induction under glutamine-deprived conditions, we aimed to determine whether TRAIL-R2/DR5 was nonetheless required for IL-8 expression. To this end, we generated HCT116 cells with stable TRAIL-R2/DR5 knockdown using two distinct shRNA sequences and analyzed IL-8 mRNA expression upon glutamine deprivation. As shown in Fig. 2A, IL-8 induction was markedly inhibited in TRAIL-R2/DR5-knockdown cells. In contrast, silencing TRAIL-R1/DR4 expression, whose protein levels remain unchanged under glutamine-limiting conditions [16], did not inhibit IL-8 upregulation upon glutamine starvation (Fig. 2B). On the contrary, the data shown in Fig. 2B reveal an increased IL-8 response in TRAIL-R1/DR4 knockdown cells. Although the precise molecular mechanism underlying this upregulation remains unclear, it is plausible that TRAIL-R2/DR5 and TRAIL-R1/DR4 may form a heterodimer that is less active than the TRAIL-R2/DR5 homodimer, and may even exert an inhibitory effect on its signaling function [44]. Accordingly, silencing TRAIL-R1/DR4 expression could favor the formation of TRAIL-R2/DR5 homodimers while limiting the assembly of less active heterodimeric complexes.

### Caspase inhibition–induced suppression of apoptosis amplifies the proinflammatory signaling cascade responsible for IL-8 upregulation under glutamine-limiting conditions

To rule out the possibility that the inflammatory response induced by glutamine starvation was a consequence of cell death [16], HCT116 cells were cultured under glutamine-deprived conditions in the presence of the pan-caspase inhibitor Q-VD-OPh (Q-VD). As shown in Fig. 2C (left panel), caspase inhibition failed to block IL-8 mRNA expression in response to glutamine deprivation despite completely preventing apoptotic cell death (Fig. 2C, right panel). The results shown in Fig. 2C demonstrate that Q-VD not only failed to inhibit IL-8 expression in response to glutamine starvation in HCT116 cells, but actually enhanced its induction (Fig. 2C, left panel). Moreover, IL-8 upregulation in response to glutamine starvation was also observed in other glutamine-dependent cancer cell lines treated with Q-VD (Fig. S1A). To assess whether Q-VD influences signaling pathways induced upon glutamine deprivation, we first analyzed activation of the integrated stress response (ISR). Although no significant changes were observed in phosphorylated eIF2α (p-eIF2α) levels, Q-VD treatment resulted in a significant increase in ATF4 expression in glutamine-deprived HCT116 cells (Fig. S1B). While the underlying mechanism remains to be elucidated, a plausible explanation is that Q-VD-mediated inhibition of apoptosis extends cell viability under glutamine-starvation, thereby providing additional time for the enhanced translation of ATF4 mRNA. Given that ATF4 is a key transcription factor driving IL-8 gene expression [32], this may account for the elevated IL-8 mRNA levels observed in the presence of Q-VD.

In this study, we initially demonstrated that IL-8 induction under glutamine deprivation is dependent on GCN2 and ATF4, but occurs independently of CHOP (Fig. 1C). To validate these findings under conditions of caspase inhibition, we conducted similar experiments in the presence of Q-VD. The results shown in Figures S1C and S1D further support the activation of a GCN2/ATF4-dependent, but CHOP-independent, signaling pathway that drives IL-8 upregulation in glutamine-deprived HCT116 tumor cells. The involvement of ATF4 in glutamine starvation-induced IL-8 expression was further confirmed in the glutamine-addicted RKO colorectal cancer cell line [37] under conditions of apoptosis inhibition (Fig. S2A).

### IL-8 upregulation under glutamine restriction depends on TRAIL-R2/DR5-mediated NF-kB activation

**Fig. 3:**
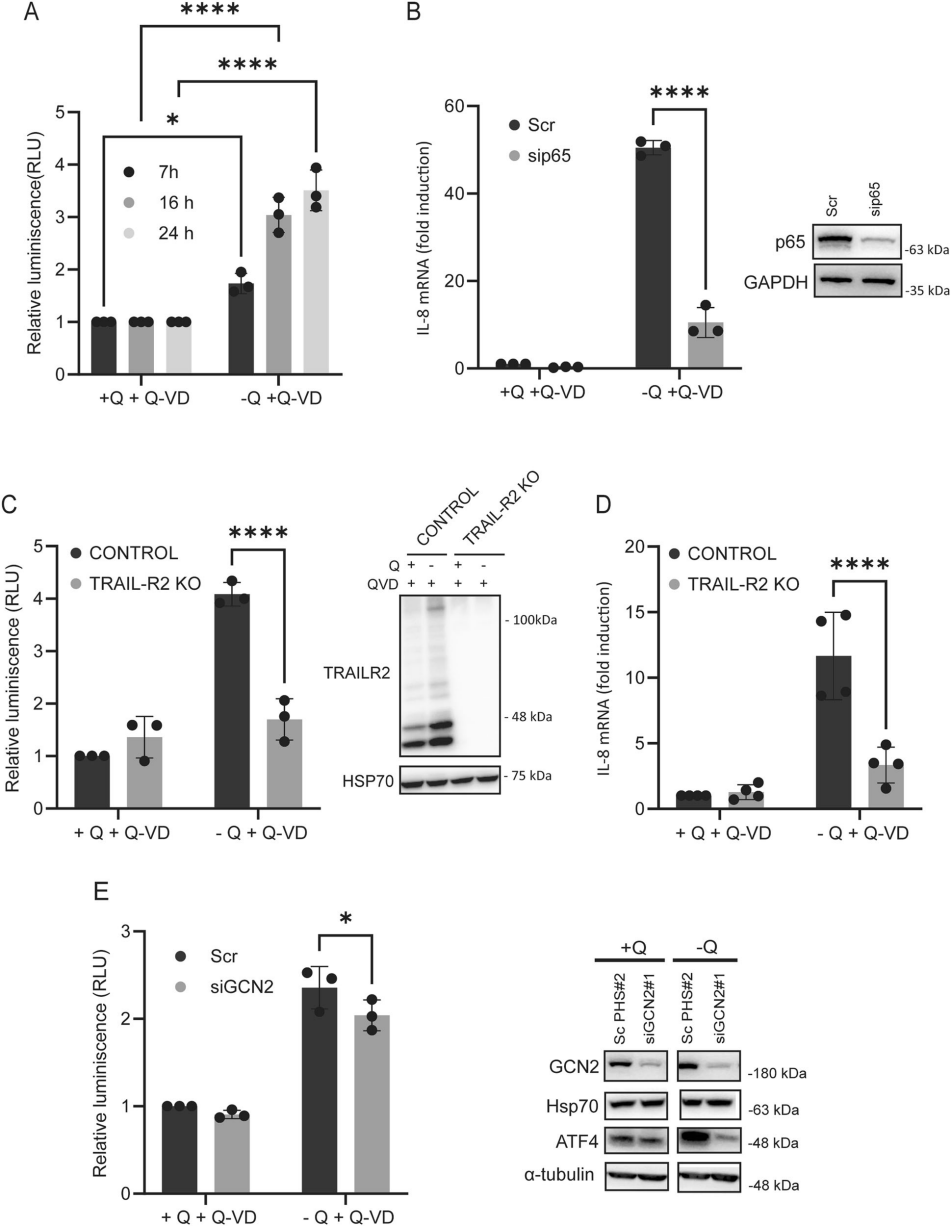
Glutamine restriction-induced upregulation of IL-8 is dependent on NF-κB activation mediated by TRAIL-R2/DR5 signaling. **A** To evaluate NF-κB transcriptional activity, HCT116 cells were transfected with 200 ng of the pSI-Check2-RLuc-NF-κB-Firefly reporter plasmid 24 h prior to treatment. Luciferase activity was assessed at the indicated time points following glutamine deprivation in the presence of 20 µM Q-VD. Relative luciferase units (RLUs) were normalized to those of control cells maintained in glutamine-replete conditions and treated with Q-VD. **B** HCT116 cells were transfected with scrambled (Scr) or p65-targeting siRNA for 48 h before treatment. The cells were deprived of glutamine in the presence of 20 µM Q-VD for 16 h. IL-8 mRNA levels were assessed via RT-qPCR, and relative IL-8 mRNA levels were determined in comparison to those of the scrambled cells treated with glutamine and Q-VD. p65 knockdown in whole-cell extracts was confirmed by western blotting. **C** To determine the role of TRAIL-R2 on NF-κB transcriptional activity following glutamine deprivation, HCT116 control and TRAILR2-KO cells were transfected as described in A. After 16 h of glutamine deprivation, TRAIL-R2 levels (right panel) and luciferase activity (left panel) were assessed. Relative luciferase units (RLUs) were calculated relative to control cells cultured in glutamine-replete conditions and treated with Q-VD. **D** HCT116 control and TRAIL-R2/DR5-KO cells were cultured under glutamine-replete or glutamine-deprived conditions in the presence of 20 µM Q-VD for 16 h. IL-8 mRNA levels were quantified by RT-qPCR, and relative expression was calculated in comparison to control cells maintained in glutamine-replete conditions with Q-VD treatment. **E** HCT116 cells were transfected with either a scrambled (Scr) oligonucleotide or a siRNA targeting GCN2. For the analysis of NF-κB transcriptional activity, cells were transfected as described in section A. Luciferase activity was assessed after 16 h of glutamine deprivation, and RLUs were normalized to those of scrambled control cells cultured with glutamine and Q-VD. GCN2 and ATF4 protein levels were assessed in whole-cell extracts by western blotting. α-Tubulin, Hsp70 and GAPDH were used as protein loading controls. Data are presented as mean ± SD from at least three independent experiments and were analyzed by two-way ANOVA. *P < 0.05; ****P < 0.0001.

NF-κB transcriptional activity has been reported to regulate the secretion of proinflammatory cytokines in response to death ligands or nutrient deprivation [26–28, 32]. Moreover, glutamine deprivation has been shown to induce NF-κB activation in various cell types [45, 46], although the underlying signaling mechanisms remain incompletely understood. To further investigate the regulatory mechanisms controlling IL-8 production in glutamine-deprived HCT116 cells, we assessed NF-κB activity using an NF-κB-driven luciferase reporter assay. As shown in Fig. 3A, culturing HCT116 cells in the absence of glutamine resulted in a significant increase in NF-κB activity between 7 and 24 hours after glutamine deprivation. Importantly, silencing p65, a key subunit of the canonical NF-κB complex, significantly reduced IL-8 mRNA upregulation in glutamine-starved HCT116 (Fig. 3B) and RKO cells (Fig. S2B), indicating that this pathway plays a crucial role in IL-8 induction under glutamine-limiting conditions. Various forms of cellular stress, including endoplasmic reticulum (ER) stress and glucose starvation, have been shown to trigger inflammatory responses through TRAIL receptor activation [25, 28]. To determine whether TRAIL-R2/DR5 contributes to the NF-κB activation observed in glutamine-deprived cells, we employed HCT116 TRAIL-R2/DR5 knockout (TRAIL-R2/DR5 KO) cells generated via CRISPR/Cas9-mediated genome editing (Fig. 3C, right panel). Compared to control cells, TRAIL-R2/DR5 KO cells exhibited a marked suppression of both NF-κB activity (Fig. 3C, left panel) and IL-8 induction (Fig. 3D) in response to glutamine deprivation. These findings support a model in which TRAIL-R2/DR5-dependent NF-κB activation is essential for IL-8 upregulation during metabolic stress induced by glutamine starvation. Importantly, this pathway functions independently of GCN2 signaling, as GCN2 silencing only modestly decreased NF-κB activity during glutamine deprivation (Fig. 3E).

### NF-kB activation and IL-8 induction upon glutamine deprivation require caspase-8 and FADD

**Fig. 4:**
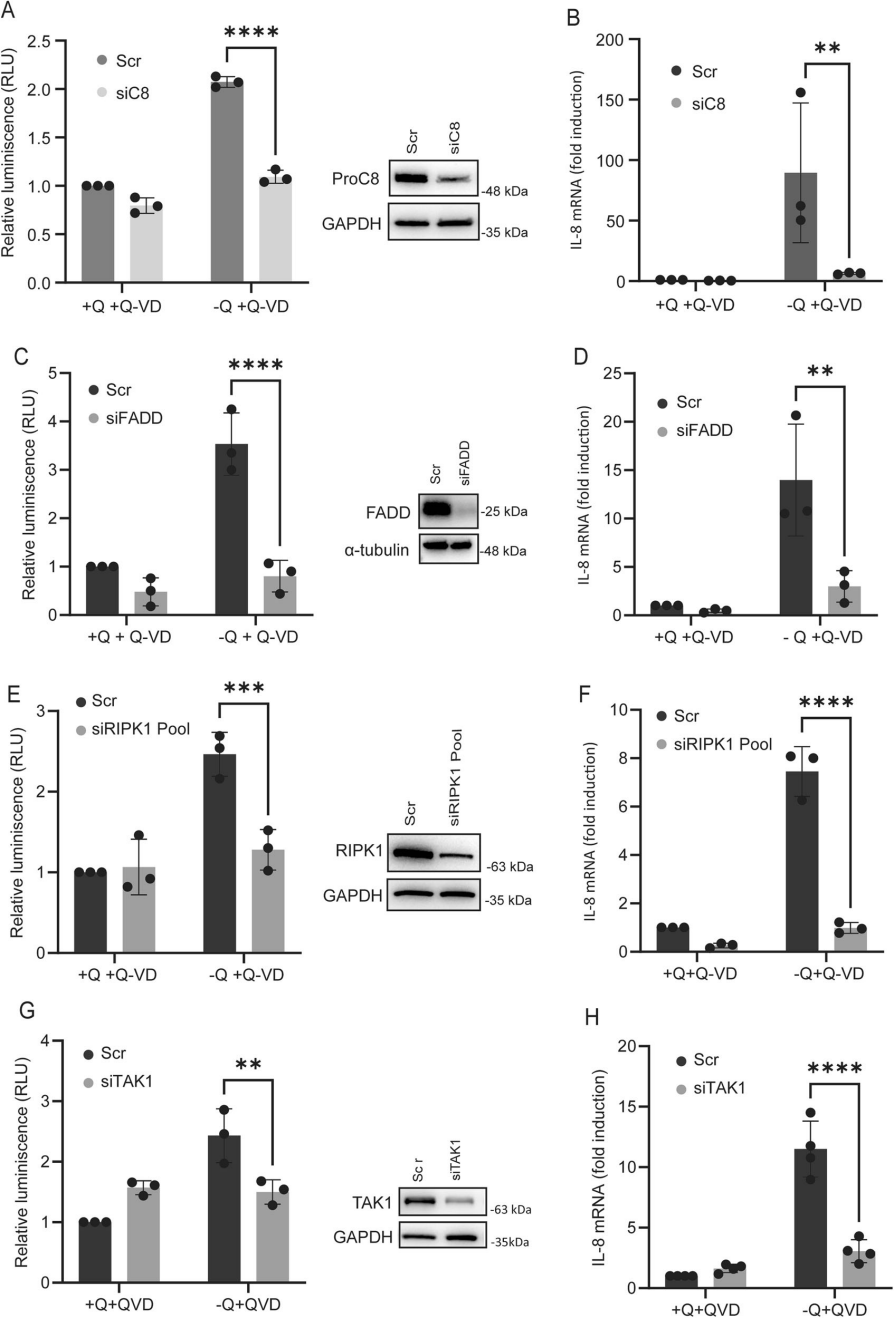
Caspase-8, FADD, RIPK1 and TAK1 are required for NF-κB activation and subsequent IL-8 induction under glutamine-deprived conditions. HCT116 cells were transfected with either a scrambled (Scr) oligonucleotide or siRNA targeting (**A, B**) caspase-8 (C8), (**C, D**) FADD, (**E,** **F**) RIPK1 or (**G, H**) TAK1 for 48 hours prior to treatment. For the analysis of NF-κB transcriptional activity, the cells were transfected with 200 ng of the pSI-Check2-RLuc-NF-κB-Firefly plasmid 24 h before treatment. After 16 h of glutamine deprivation in the presence of 20 μM Q-VD, luciferase activity was measured, and relative luciferase units (RLUs) were calculated in comparison to those of scrambled cells supplemented with glutamine. Protein knockdown in whole-cell extracts was confirmed by western blotting. GAPDH and α-Tubulin levels were used as a normalization control. IL-8 mRNA levels were measured by RT-qPCR after 16 h of treatment, and the relative levels of IL-8 mRNA were normalized to those in scrambled cells treated with glutamine and Q-VD. The data are presented as mean ± SD from at least three independent experiments and were analyzed by two-way ANOVA. ns not statistically significant, **P < 0.01; ****P < 0.0001.

Caspase-8 can function as a scaffold protein, assembling an intracellular signaling complex containing FADD, caspase-8, and RIPK1 (also known as the FADDosome), which can promote inflammatory cytokine production downstream of TRAIL receptor activation [27], even in the absence of its ligand TRAIL [24, 28]. To evaluate the role of caspase-8 in the inflammatory response induced by glutamine deprivation, we first silenced caspase-8 expression using siRNA and examined NF-κB activation. Caspase-8 knockdown significantly reduced NF-κB activation under glutamine-deprived conditions (Fig. 4A and S2C). Moreover, caspase-8-deficient cells exhibited a marked inhibition of IL-8 mRNA upregulation upon glutamine starvation (Fig. 4B and S2D). Our results also reveal that the adaptor protein FADD is essential for both NF-κB activation and IL-8 induction in cancer cells experiencing metabolic stress due to glutamine starvation, as FADD silencing by siRNA significantly suppressed both processes (Fig. 4C, D). Collectively, these findings indicate that the proinflammatory response triggered by glutamine deprivation depends on TRAIL-R2/DR5, caspase-8, and FADD, highlighting a role for key components of the extrinsic apoptotic pathway in this inflammatory process. Next, we sought to investigate the potential involvement of the TRAIL ligand in IL-8 upregulation under glutamine-starved conditions. Glutamine deprivation-induced apoptosis in various cancer cell lines occurs by a TRAIL-R2/DR5-dependent and TRAIL-independent mechanism [16]. To assess whether TRAIL contributes to IL-8 induction, we employed a soluble chimeric decoy receptor (TRAIL-R2/DR5-Fc) that neutralizes extracellular TRAIL-induced apoptosis signaling (Fig. S2E, left panel). Notably, TRAIL-R2/DR5-Fc treatment did not attenuate IL-8 induction in HCT116 cells following glutamine deprivation (Fig. S2E, right panel). Given that HCT116 cells lack detectable levels of endogenous TRAIL expression [16], yet still exhibit robust IL-8 mRNA upregulation upon glutamine withdrawal, altogether these findings suggest that IL-8 induction occurs through a TRAIL ligand-independent activation of proinflammatory signaling in response to metabolic stress.

### RIPK1 and TAK1 are essential for NF-κB activation and IL-8 induction under glutamine deprivation

In addition to FADD and caspase-8, the intracellular FADDosome complex includes RIPK1 kinase, a critical regulator of inflammatory gene expression [47, 48]. To investigate the role of RIPK1 in NF-κB activation and IL-8 upregulation following glutamine deprivation, we silenced RIPK1 expression in HCT116 cells prior to glutamine withdrawal. As shown in Fig. 4E, F, RIPK1 knockdown markedly suppressed both NF-κB activity and IL-8 mRNA expression under glutamine-starved conditions, indicating that RIPK1 is essential for mediating the inflammatory response to this metabolic stress in HCT116 cells.

TGF-β-activated kinase 1 (TAK1) is a critical mediator of NF-κB signaling following TNFR1 activation by TNF-α [49], engagement of TRAIL receptors by TRAIL [27], and under conditions of cellular stress [28]. To investigate the role of TAK1 in the inflammatory response to glutamine deprivation, TAK1 expression was silenced in HCT116 cells prior to glutamine limitation. As shown in Fig. 4G, H, TAK1 knockdown markedly attenuated NF-κB activation and IL-8 up-regulation under glutamine-starved conditions. Collectively, these findings support a model in which caspase-8, FADD, RIPK1, and TAK1 function as key downstream effectors required for NF-κB activation and IL-8 induction following TRAIL-R2/DR5 activation in response to glutamine deprivation–induced metabolic stress in HCT116 cells.

### Role of cFLIP in NF-κB activation and IL-8 induction under glutamine-deprived conditions

**Fig. 5:**
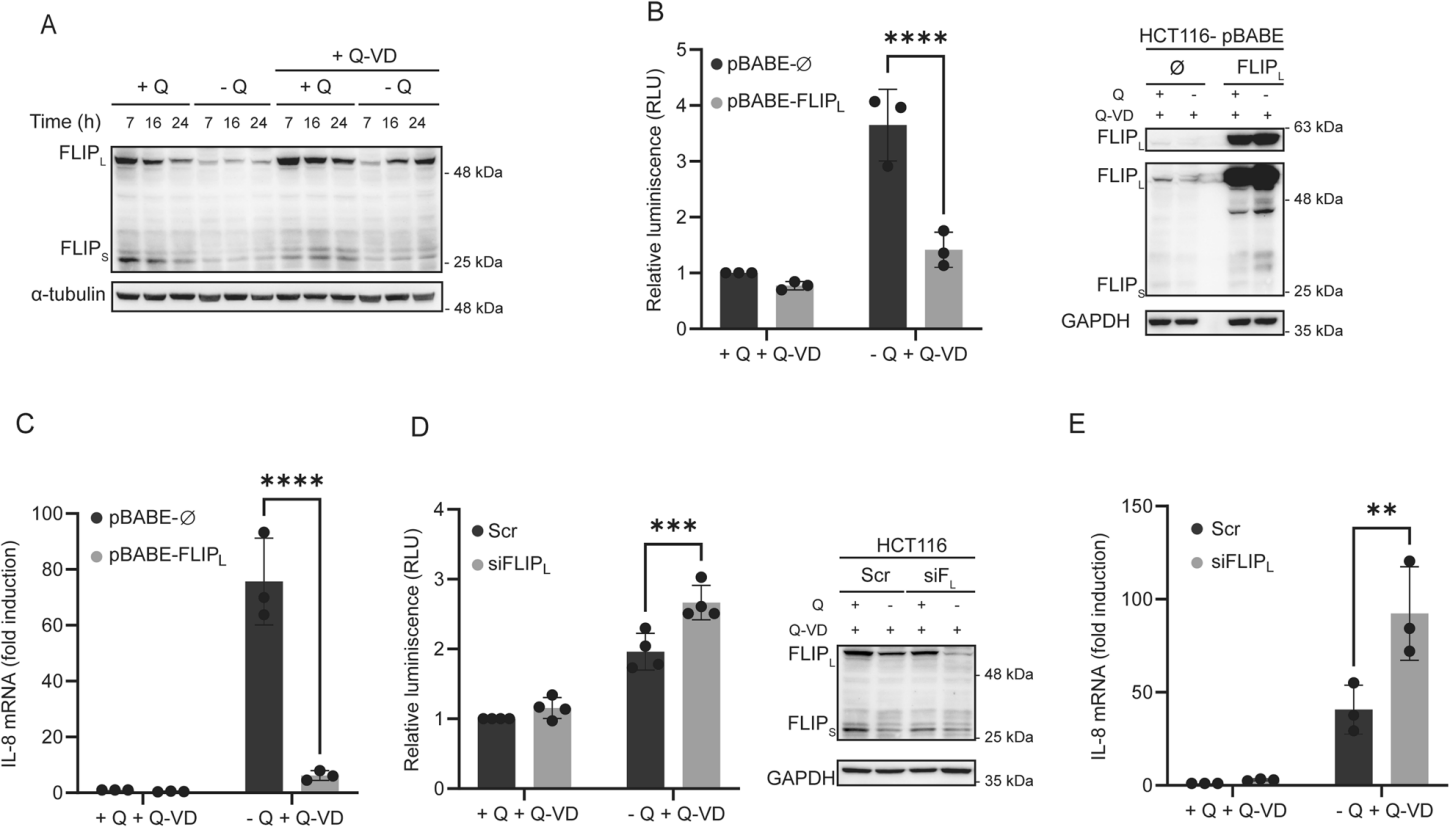
Role of cFLIP in NF-kB activation and IL-8 induction upon glutamine deprivation. **A** HCT116 cells were deprived of glutamine for the indicated time periods in the presence or absence of Q-VD (20 µM). cFLIP_L_ and cFLIP_S_ levels were analyzed in whole-cell extracts by western blotting. **B** To analyze NF-κB transcriptional activity, HCT116 pBABE-ø and pBABE-FLIP_L_ cells were transfected with 200 ng of the pSI-Check2-RLuc-NF-κB-Firefly plasmid 24 h prior to treatment. After 16 h of glutamine deprivation with 20 µM Q-VD, luciferase activity was measured, and relative luciferase units (RLUs) were calculated in comparison to those of pBABE-ø cells treated with glutamine and Q-VD. cFLIP levels in whole-cell extracts were assessed by western blotting. **C** HCT116 pBABE-ø and pBABE-FLIP_L_ cells were either deprived or not deprived of glutamine in the presence of 20 µM Q-VD for 16 h. IL-8 mRNA levels were assessed by RT-qPCR. Relative IL-8 mRNA levels were determined in comparison to those of pBABE-ø cells treated with glutamine and Q-VD. **D** HCT116 cells were transfected with scrambled (Scr) or a siRNA targeting cFLIP_L_ (siF_L_) for 48 h prior to treatment. To test NF-κB transcriptional activity, cells were transfected as described in B. cFLIP_L_ knockdown in whole-cell extracts was confirmed by western blotting. **E** HCT116 cells were transfected as described in D for 48 h prior to treatment. Cells were either deprived or not deprived of glutamine in the presence of 20 µM Q-VD for 16 h. IL-8 mRNA levels were assessed by RT-qPCR. Relative IL-8 mRNA levels were determined in comparison to those of scrambled cells treated with glutamine and Q-VD. The data are presented as mean ± SD from at least three independent experiments and were analyzed by two-way ANOVA. **P < 0.01; ***P < 0.001; ****P < 0.0001.

The antiapoptotic proteins cFLIP_L_ and cFLIP_S_ are key regulators of caspase-8 activation upon TRAIL receptor stimulation by TRAIL [50–52]. More recently, cFLIP_L_ has been identified as a negative regulator of Fas- and TRAIL-induced inflammatory cytokine production [24]. While cFLIP is a well-established NF-κB transcriptional target [53], its role in modulating NF-κB signaling remains controversial [24, 54–56]. As shown in Fig. 5A, both cFLIP_L_ and cFLIP_S_ were markedly downregulated in HCT116 cells following glutamine deprivation, although partial recovery was observed over time in the presence of Q-VD. A marked decrease in cFLIP_L_ levels was also detected in RKO colon cancer cells under glutamine-limiting conditions (Fig. S3A left panel). To assess the functional relevance of cFLIP_L_ downregulation in NF-κB activation and IL-8 induction, we ectopically expressed cFLIP_L_ in HCT116 and RKO cells via retroviral transduction and subsequently evaluated NF-κB activity and IL-8 mRNA expression levels. As shown in Fig. 5B, cFLIP_L_ overexpression markedly suppresses NF-κB activation under glutamine-limiting conditions. Furthermore, IL-8 mRNA induction was significantly inhibited in cFLIP_L_-overexpressing cells (Fig. 5C and S3A right panel). Conversely, siRNA-mediated knockdown of cFLIP_L_ enhanced both NF-κB activity and IL-8 expression under glutamine-deprived conditions (Fig. 5D, E). In contrast, silencing of cFLIP_S_ produced the opposite effect, resulting in decreased NF-κB activity (Fig. S3B) and partial attenuation of IL-8 induction (Fig. S3C). Collectively, these findings suggest that cFLIP_L_ and cFLIP_S_ play opposing regulatory roles in modulating NF-κB signaling and IL-8 expression downstream of TRAIL-R2/DR5 activation in glutamine-starved colon cancer cells.

### α-Ketoglutarate inhibits IL-8 upregulation triggered by glutamine starvation

**Fig. 6:**
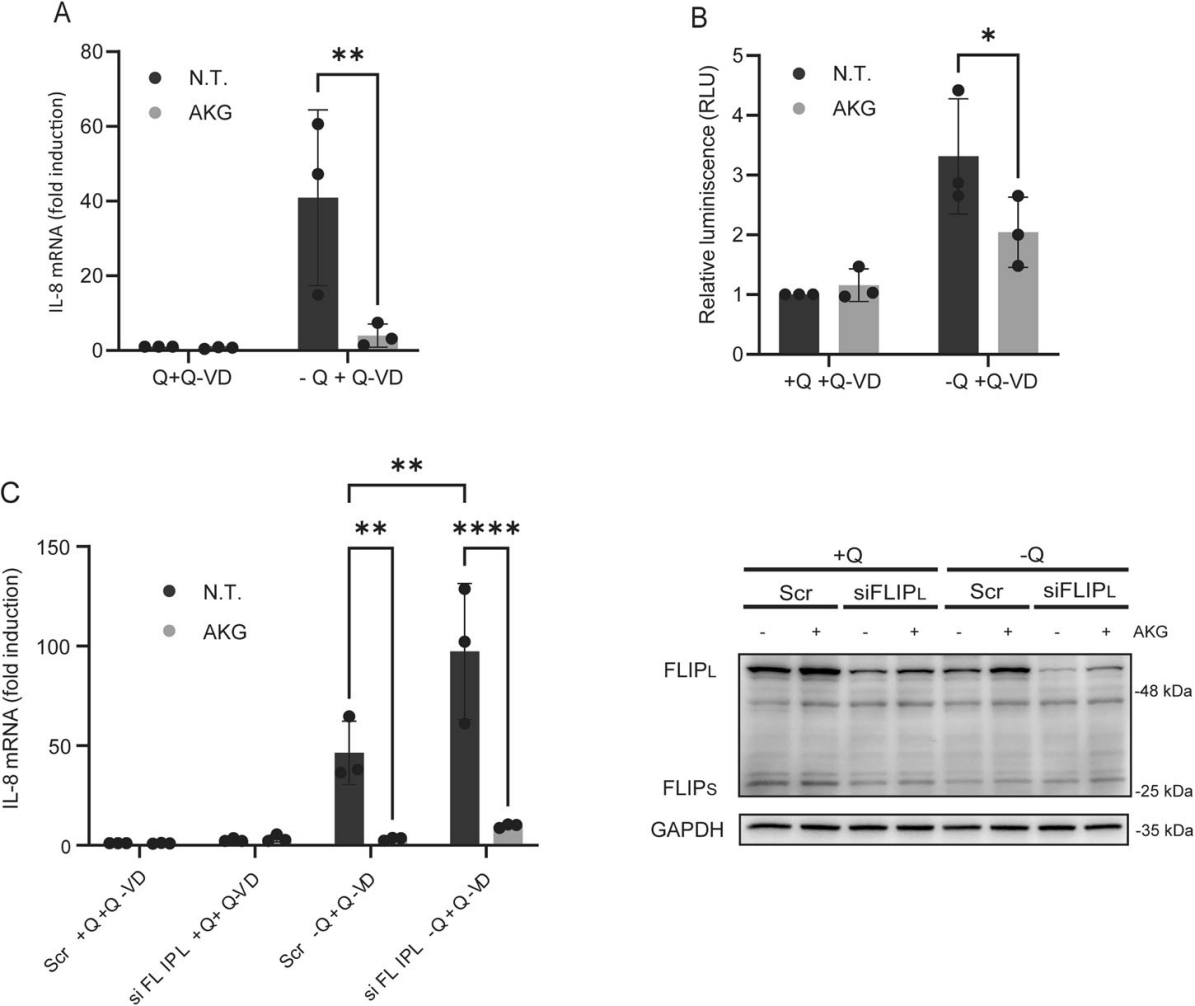
Role of AKG in IL-8 induction and NF-kB activation upon glutamine deprivation. **A** HCT116 cells were cultured in the presence or absence of glutamine, with or without 20 µM Q-VD. Where indicated, 5 mM dmAKG was added to the culture medium. After 16 h of treatment, IL-8 mRNA levels were assessed by RT-qPCR. Relative IL-8 mRNA levels were determined in comparison to those in untreated (N.T.) cells treated with glutamine and Q-VD. **B** To analyze NF-κB transcriptional activity, HCT116 cells were transfected with 200 ng of the pSI-Check2-RLuc-NF-κB-Firefly plasmid 24 hours prior to treatment (as described in **A**). Luciferase activity was measured, and relative luciferase units (RLUs) were calculated in comparison to those in N.T. cells treated with glutamine. In **C** HCT116 cells were transfected with either a scrambled (Scr) oligonucleotide or a siRNA oligonucleotide targeting cFLIP_L_ for 48 h prior to treatment. Cells were either deprived or not deprived of glutamine in the presence of 20 µM Q-VD for 16 h, and treated with 5 mM dmAKG where indicated. IL-8 mRNA levels were assessed by RT-qPCR. Relative IL-8 mRNA levels were normalized to those of scrambled control cells cultured in glutamine-containing medium and treated with Q-VD. cFLIP protein levels were assessed by western blotting.

α-ketoglutarate (AKG), a central metabolite in glutamine catabolism, prevents both cFLIP_L_ downregulation and cell death in glutamine-deprived HCT116 cells [16]. To investigate whether AKG also modulates IL-8 expression under glutamine-limiting conditions, HCT116 cells were treated with the cell-permeable AKG analog dimethyl-α-ketoglutarate (dmAKG) in the presence or absence of glutamine. As shown in Fig. 6A, dmAKG treatment markedly suppressed IL-8 upregulation induced by glutamine deprivation. Next, we investigated whether dmAKG also suppresses NF-κB activation under glutamine-deprived conditions. As shown in Fig. 6B, dmAKG supplementation significantly reduced NF-κB activity in glutamine-starved HCT116 cells.

Given that α-ketoglutarate regulates cFLIP_L_ levels [16], and that both dmAKG and cFLIP_L_ appear to influence NF-κB activation and IL-8 expression, we next investigated whether the inhibitory effect of dmAKG on IL-8 expression is primarily mediated through cFLIP_L_ stabilization. To address this question, cFLIP_L_ protein levels and IL-8 mRNA up-regulation were assessed under glutamine-deprived conditions in both control and cFLIP_L_-knockdown cells, in the presence or absence of dmAKG (Fig. 6C). Although dmAKG did not fully restore cFLIP_L_ levels in knockdown cells, it still suppressed IL-8 induction in both control and knockdown conditions. These findings suggest that while cFLIP_L_ stabilization may contribute to the inhibitory effect of dmAKG on IL-8 upregulation, additional cFLIP_L_-independent mechanisms are likely involved.

Collectively, our findings suggest that glutamine deprivation, through cFLIP_L_ downregulation, facilitates TRAIL-R2/DR5-mediated NF-κB activation, which, in concert with the GCN2/ATF4 signaling pathway, drives IL-8 gene expression in human colon cancer cells. Moreover, AKG controls NF-κB activation and IL-8 upregulation, at least in part by stabilizing cFLIP_L_ levels, thereby providing a metabolic checkpoint that limits the inflammatory response induced by glutamine starvation.

## Discussion

While cancer cells often rely on glutamine metabolism for survival and proliferation [37, 57], the impact of glutamine deficiency on inflammatory responses remains underexplored. Our study provides novel evidence that IL-8 expression and secretion in response to glutamine deprivation in glutamine-dependent colon cancer cells require the activation of two distinct signaling pathways: the GCN2-mediated Integrated Stress Response (ISR) and the NF-kB pathway. The latter is activated through a TRAIL-R2/FADD/caspase-8-mediated signaling pathway that involves the protein kinases RIPK1 and TAK1. Furthermore, our findings reveal that cFLIP_L_ functions as a potent inhibitor of IL-8 production upon TRAIL-R2/DR5 activation under glutamine-restricted conditions.

GCN2 is a key kinase that enables cellular adaptation to metabolic stress caused by amino acid deprivation by regulating both protein synthesis and amino acid metabolism [12, 58, 59]. Under amino acid-limited conditions, GCN2 activation promotes ATF4 translation, which in turn regulates genes involved in cellular stress responses [38], apoptosis [16], and immune-driven cytokine production [60]. However, GCN2 has also been implicated in suppressing innate immune responses [17, 61]. Our findings indicate that glutamine deprivation induces IL-8 gene expression and secretion in glutamine-addicted colon cancer cells via the GCN2/ATF4 pathway. Given that the IL-8 promoter contains ATF4 binding sites, it is plausible that ATF4 directly regulates IL-8 transcription, consistent with findings in glucose-starved cells [32]. Although activation of the GCN2/ATF4/CHOP pathway driven by metabolic stress leads to the upregulation of TRAIL-R2/DR5, which is critical for apoptosis induction [16], our data reveal that CHOP induction and TRAIL-R2 upregulation are not essential for IL-8 up-regulation under glutamine-restricted conditions. These findings suggest that IL-8 induction under glutamine deprivation is primarily mediated by the GCN2/ATF4 signaling pathway, with ATF4 likely acting as a key transcriptional regulator of IL-8 gene expression.

TRAIL-R2/DR5 activation has been reported in response to various stress conditions [16, 28, 40, 42]. While TRAIL-R2/DR5 plays a pivotal role in IL-8 induction in glutamine-starved tumor cells, our data indicate that its upregulation following ISR activation is not required for IL-8 synthesis. Notably, in this context, the TRAIL receptor-mediated inflammatory response appears to be ligand-independent, as previously demonstrated under other stress conditions [40, 42, 62]. One possible explanation for ligand-independent IL-8 induction upon glutamine deprivation is that ER stress, which can be triggered under glutamine limitation [33], may sequester TRAIL receptors within the ER-Golgi compartment, leading to their autoactivation by misfolded proteins [63]. Alternatively, TRAIL-R2/DR5 may contribute to IL-8 production independently of its expression levels, as observed in non-small cell lung carcinoma under basal conditions [25].

Glutamine deprivation has been shown to activate NF-kB signaling in multiple cell types [45, 46]. Our findings confirm that NF-kB activation occurs in colon cancer cells under glutamine restriction and that the canonical NF-kB subunit p65 is required for IL-8 induction in this context. Under certain stress conditions, such as ER stress, NF-kB activation relies on the formation of the intracellular FADDosome complex, composed of FADD, caspase-8, and RIPK1, which is triggered by TRAIL-R2/DR5 [28]. As expected, transient knockdown experiments corroborated the requirement of caspase-8, FADD, RIPK1, and TAK1 for NF-κB activation and IL-8 induction in response to glutamine deprivation. However, in the absence of an exogenous ligand, FADDosome formation under glutamine starvation may proceed too slowly to be detected experimentally.

Our study also highlights a critical role for cFLIP in modulating NF-kB activation and IL-8 induction during glutamine deprivation. cFLIP is a conserved regulatory protein that mainly exists in two functional isoforms, cFLIP_L_ and cFLIP_S_, both of which inhibit death receptor-mediated apoptosis [51, 64]. While cFLIP_L_ can paradoxically facilitate apoptosis under certain conditions [65], it is also implicated in the regulation of necroptosis [66] and in the formation of the ripoptosome, a death receptor-independent apoptotic platform [67, 68]. More recently, cFLIP_L_ was shown to inhibit Fas- and TRAIL-induced NF-kB activation by interfering with FADDosome assembly [24]. Our data reveal that cFLIP_L_ functions as a potent suppressor of NF-kB activation and IL-8 synthesis under glutamine-starved conditions, whereas cFLIP_S_ seems to exert an opposing effect, similar to its function in ripoptosome regulation [67]. Considering that HCT116 cells exhibit higher cFLIP_L_ than cFLIP_S_ expression, the observed reduction in cFLIP_L_ upon glutamine deprivation likely plays a more significant role in promoting the formation of the signaling complex. When cFLIP_S_ levels are also reduced, the altered cFLIP_L_/cFLIP_S_ ratio may shift in favor of cFLIP_L_, thereby hindering the formation of the intracellular complex necessary for NF-κB activation. Since TRAIL-R2/FADD/pro-caspase-8 and cFLIP colocalize in intracellular membrane compartments even in the absence of TRAIL [69], cFLIP_L_ downregulation in glutamine-dependent tumor cells could facilitate TRAIL-R2/DR5 aggregation and subsequent NF-kB activation, leading to IL-8 induction. Our data also suggest that when apoptosis induced by glutamine deprivation is inhibited, NF-κB activation may progressively upregulate cFLIP_L_ expression over time [53], potentially serving as a control mechanism for the pro-inflammatory response, which warrants further investigation.

**Fig. 7:**
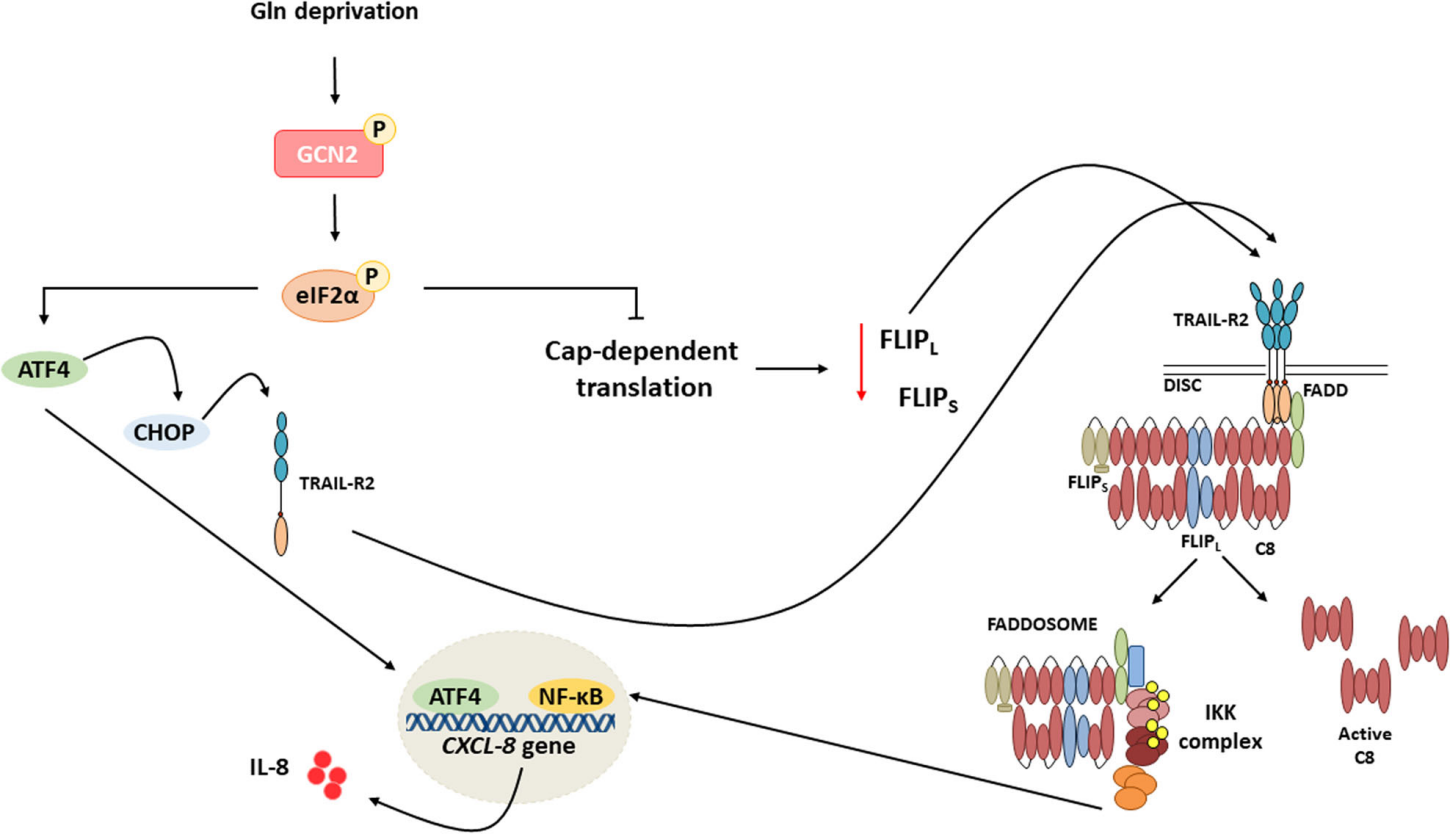
Schematic diagram of the signaling pathways activated by glutamine deprivation that may contribute to IL-8 induction in HCT116 cells. Glutamine deprivation activates the GCN2–ATF4 pathway while concurrently reducing cFLIP_L_ levels. This reduction, in conjunction with TRAIL-R2/DR5 activation, promotes NF-κB signaling, which, together with ATF4, drives IL-8 gene expression.

Taken together, our findings suggest potential molecular mechanisms that may underlie the proinflammatory response observed upon glutamine deprivation in glutamine-dependent tumor cells. Two distinct pathways contribute to IL-8 induction under these conditions (Fig. 7): activation of GCN2/ATF4 signaling, likely functioning at the transcriptional level, and the concurrent downregulation of cFLIP_L_ resulting from reduced cellular AKG levels. This decrease, in conjunction with TRAIL-R2/DR5 aggregation, may ultimately facilitate FADDosome formation and NF-κB activation, which synergize with ATF4 in driving IL-8 production. In the context of solid tumors, nutrient and oxygen deprivation in regions distant from blood vessels can lead to cellular stress and death. However, an inflammatory response in these regions may promote angiogenesis and tumor expansion, thereby exacerbating disease progression.

## Materials and methods

### Cell culture

The HEK293 and HCT116 cell lines were kindly provided by Dr. A. Rodriguez (Universidad Autónoma Madrid, Spain) and Dr. J.A. Pintor-Toro (CABIMER, Seville, Spain), respectively. The human colorectal carcinoma cell line RKO was purchased from Cell Lines Service GmbH (Eppelheim, Germany). MDA-MB468 cells were a gift from Dr. J. Arribas (VallD’Hebron Institute of Oncology, Barcelona, Spain). U2OS human osteosarcoma cell line was provided by Dr. Pablo Huertas (CABIMER, Seville, Spain). HCT116 TRAIL-R2 KO cells were a donation from Dr. Markus Rehm (University of Stuttgart, Germany). HEK293 and MDA-MB468 cells were maintained in DMEM supplemented with 10% foetal bovine serum, 2 mM L-glutamine, 50 U of penicillin/ml and 50 µg of streptomycin/ml. HCT116 and RKO cells were cultured in McCoy’s medium supplemented with 10% foetal bovine serum, 2 mM L-glutamine, 50 U of penicillin/ml and 50 µg streptomycin/ml. The cells were grown at 37 °C in a 5% CO_2_-humidified, 95% air incubator. The cell lines were regularly tested for mycoplasma contamination. Glutamine deprivation experiments were performed in high-glucose DMEM lacking NEAAs supplemented with 10% dialyzed foetal bovine serum, penicillin (50 U/ml), and streptomycin (50 μg/ml).

### Reagents and antibodies

Media supplements and chemical reagents for molecular biology and buffer preparation were obtained from Sigma‒Aldrich (St. Louis, MO, USA). The anti-Hsp70 (H5147) antibody, propidium iodide, dimethyl α-ketoglutarate and puromycin were obtained from Sigma‒Aldrich. The anti-pro-caspase-8 antibody (04-574) was obtained from Upstate Millipore (NY, USA). Anti-α-tubulin (SC-23948), anti-GAPDH (SC-47724), anti-GCN2 (SC-374609) and anti-p65 (SC-8008) antibodies were purchased from Santa Cruz (CA, USA). The anti-ATF4 antibody (10835-1-AP) was purchased from Proteintech (IL, USA). Anti-TRAIL-R1 (AF347), anti-TRAIL-R2/DR5 (AF631) and recombinant TRAIL-R2-Fc chimeric protein (631-T2) were obtained from R&D Systems (Minneapolis, USA). The anti-c-FLIP monoclonal antibody 7F10 (ALX-804-961-0100) was obtained from Enzo Life Sciences (NY, USA). Anti-p-eIF2α (S51) (119A11) (3597), anti-eIF2α (D7D3) (5324), anti-CHOP (D46F1) (5554) and anti-TAK1 (4505) antibodies were purchased from Cell Signaling Technology (CA, USA). Anti-FADD (610399) and anti-RIPK1 (610458) antibodies were obtained from BD Biosciences (NJ, USA). Horseradish peroxidase-conjugated secondary antibodies were obtained from Dako (P0447, P0448, P0449) (Cambridge, UK). Recombinant human TRAIL was produced as previously described [70]. Q-VD-OPh was obtained from AppexBio (Houston, USA).

### ELISA assay

Supernatants were collected and centrifuged (3000 g, 5 min) to eliminate dead cells and cellular debris and then stored at −80 °C. When necessary, the supernatants were diluted to achieve the optimal optical range and analysed via DuoSet ELISA Development Systems according to the manufacturer’s guidelines. Optical densities were measured with a Thermo Fisher Scientific Varioskan lux microplate spectrophotometer at wavelengths of 450 and 540 nm. The final cytokine concentrations (pg/ml) were normalized to the protein concentration, which was calculated after the protein content in the cells attached to the plate was measured and is presented as pg/mg of protein in the figures.

### Real-time qPCR

RNA was extracted using PRImeZOL (Canvax Biotech Córdoba, Spain) following the manufacturer’s instructions and reverse-transcribed using an iScript cDNA synthesis kit (Bio-Rad, ref. 1708891). mRNA expression was analysed in triplicate via RT‒qPCR on an ABI Prism7500 sequence detection system with SYBR Green reagent (Bio-Rad, ref. 1725124). Glyceraldehyde-3-phosphate dehydrogenase (GAPDH) was used as an internal control. Alternatively, M-MLV reverse transcriptase (Invitrogen, Carlsbad, CA, USA) was used to reverse transcribe total RNA, and mRNA expression was assessed via qPCR using predesigned assay-on-demand primers and probes (Applied Biosystems). Hypoxanthine-guanine phosphoribosyltransferase (HPRT1) was used as an internal control.

The primers for SYBR Green analysis were as follows:

**Table:** 

GAPDH	Forward: 5′-ATGGGGAAGGTGAAGGTCG-3′ Reverse: 5′-GGGTCATTGATGGCAACAATATC-3′
IL-8/CXCL8	Forward: 5′-CTGCGCCAACACAGAAATTATTGTA-3′ Reverse: 5′-TTCACTGGCATCTTCACTGATTCTT-3′

TaqMan primers and probes:

**Table:** 

HPRT	Hs01003267_m1
IL-8 CXCL8	Hs00174103_m1
TRAIL-R2/DR5 TNFSF10	Hs00366278_m1

### Immunoblot analysis of proteins

Cells were washed with phosphate-buffered saline (PBS) and lysed in TR3 buffer (10 mM Na_2_HPO_4_, 10% glycerol, 3% SDS). The protein content was measured with the DC (detergent compatible) protein assay reagent (Bio-Rad Laboratories, USA) before the addition of Laemmli sample buffer. Proteins were resolved on SD‒polyacrylamide minigels and detected as described previously [69]. α-Tubulin, Hsp70 and GAPDH were used as protein loading controls.

### RNA interference

siRNAs against GCN2, ATF4, CHOP, TRAIL-R1, p65, caspase-8, FADD, RIPK1, TAK1, FLIP_L_, FLIP_S_ and non-targeting scrambled oligonucleotides were synthesized by Sigma (St. Louis, MO). HCT116 cells were transfected with siRNAs using jetPRIME (Polyplus Transfection) following the manufacturer’s instructions. After 24 h, the transfection medium was replaced with regular medium, and the cells were further incubated for 24 h before further analysis. RKO cells were transfected with siRNAs using DharmaFECT-1 (Dharmacon), following the manufacturer’s protocol. Six hours post-transfection, the medium was replaced with standard growth medium, and the cells were incubated for an additional 48 h prior to further analysis.

***siRNAs:***

**Table:** 

GCN2:	5’-CAGCAGAAAUCAUGUACGAdTdT-3'
ATF4:	5’-GCCUAGGUCUCUUAGAUGAdTdT-3'
CHOP:	5’-AGGGAGAACCAGGAAACGGAA-3’ 5’-ACGGCTCAAGCAGGAAATCGA-3’ 5’-AAGGAAGTGTATCTTCATACA-3’ 5’-CAGCTTGTATATAGAGATTGT-3'
TRAIL-R1:	5’-GGAACUUUCCGGAAUGACAdTdT-3′
p65:	5’-GCCCUAUCCCUUUACGUCAdTdT-3'
Caspase-8:	5’-GGAGCUGCUCUUCCGAAUUdTdT-3'
FADD:	5’-GAUUGGAGAAGGCUGGCUCdTdT-3'
RIPK1:	5’-CCACUAGUCUGACGGAUAAdTdT-3’ 5’-UUAAGAGGUCUUCCUGCUUACGCUUdTdT-3’ 5’-GAACCCAGGGACUCAUGAUdTdT-3'
TAK1:	5’-GGAGAUCGAGGUGGAAGAGdTdT-3'
FLIP_L_:	5’-CCUAGGAAUCUGCCUGAUAdTdT-3'
FLIP_S_:	5’-CACCCUAUGCCCAUUGUCCdTdT-3'
Scrambled:	5’-UGGUUUACAUGUUGUGUGAdTdT-3'

### Retroviral and lentiviral vectors

FLIP(L) retroviral vectors for stable gene expression have been described previously [69, 71, 72]. For silencing experiments, shRNAs against TRAILR2 in a pSUPER vector (OligoEngine) were digested and cloned between the *EcoRI and ClaI* sites in an H1 promoter-driven GFP-encoding pLVTHM lentiviral vector [73]. Lentiviruses and retroviruses were produced by transfecting HEK293-T cells with the corresponding vectors via the calcium phosphate method. Lentivirus or retrovirus-containing supernatants were collected 48 h after transfection and concentrated by ultracentrifugation at 22,000 rpm for 90 min at 4 °C. Stable populations of HCT116 and RKO cell lines infected with retroviruses were obtained after selection in culture medium containing puromycin (1.5 µg/ml) for 48 h. Tumor cells infected with GFP-expressing lentiviruses were detected by flow cytometry.

***shRNA sequences:***

**Table:** 

TRAIL-R2#1:	5’GATCCCC**GACCCTTGTGCTCGTTGTC**TTCAAGAGA**GACAACGAGCACAAGGGTCT**TTTTTA3’
TRAIL-R2#2:	5’GATCCCC**TCATGTATCTAGAAGGTAA**TTCAAGAGA**TTACCTTCTAGATACATGA**TTTTTA3'
Scrambled:	5’GATCCCC**CTTTGGGTGATCTACGTTA**TTCAAGAGA**TAACGTAGATCACCCAAAG**TTTTTA3'

### Determination of apoptosis

Cells (3 × 10^5^/well) were seeded in 6-well plates as indicated in the figure legends. After treatment, hypodiploid apoptotic cells were detected by flow cytometry according to published procedures [69]. Quantitative analysis of the cell cycle and subG1 cells was carried out with a FACSCalibur cytometer using Cell Quest software (Becton Dickinson, Mountain View, CA, USA).

### Assessment of luciferase activity

A luciferase reporter with an internal control for the NF-kB signaling plasmid (a gift from Dr. Cristina Muñoz-Pinedo, Addgene plasmid #106979) was transfected into HCT116 cells using jetPRIME (Polyplus Transfection) following the manufacturer’s instructions. Twenty-four hours after transfection, the cells were cultured in the presence or absence of glutamine and 20 µM Q-VD for the indicated times before collection. Luciferase activity in the cell lysates (containing equal amounts of protein) was assessed using the Dual-Luciferase Reporter Assay System (Promega, Madison, WI, USA). Samples were analysed with a Varioskan Flash microplate reader (Thermo Electron Corporation, Waltham, MA, USA). For each experiment and condition, firefly and renilla light were detected in duplicate. The firefly luciferase activity was normalized to the Renilla activity and then to the levels of the untreated samples.

### Statistical analysis

All the data are presented as the mean ± standard deviation (SD) of at least three independent experiments. Statistical analysis was performed using GraphPAD Prism 10 (GraphPad Software, San Diego, CA, USA). The differences between groups were determined via two-way ANOVA. A *P* value < 0.05 was considered significant. *P < 0.05; **P < 0.01; ***P < 0.001; ****P < 0.0001.

## Supplementary information

Supplementary figure legends

Supplementary Figure 1

Supplementary Figure 2

Supplementary Figure 3

Original data

## Data Availability

All the data generated or analysed during this study are included in the main text and the supplementary information files.
