# Supplementary material for: Activation of the integrated stress response and loss of cFLIPL under glutamine limitation induce IL-8 gene expression and secretion in glutamine-dependent tumor cells
Source: Cell Death Discov. 2025 Jul 19;11:332. doi: 10.1038/s41420-025-02625-3 (PMC12276259; doi:10.1038/s41420-025-02625-3)
Supplement: Supplementary file 1 — Supplementary figure legends [file 41420_2025_2625_MOESM1_ESM.docx]

**Supplementary figure legends**

**Figure S1. IL-8 induction in response to glutamine deprivation occurs independently of caspase activation and CHOP expression. A)** Cells were incubated with 20 µM Q-VD in either complete or glutamine-deprived medium for 24 (RKO), 48 (MDA-MB468) or 16 (U2OS) hours. IL-8 mRNA levels were assessed by RT-qPCR as described in the Materials and Methods section. Relative mRNA levels were calculated by comparing them to those observed in cells cultured in glutamine-containing medium. **B)** HCT116 cells were subjected to glutamine deprivation for the indicated time points, either in the presence or absence of 20 µM Q-VD, and protein levels were assessed in whole-cell extracts by western blotting. GAPDH and α-tubulin were used as protein loading controls. **C)** and **D)** HCT116 cells were transfected with either a scrambled oligonucleotide (scr) or siRNAs targeting GCN2, ATF4 or CHOP. Forty-eight hours after transfection the cells were incubated for 16 hours in complete medium or glutamine-deprived medium, with or without 20 µM Q-VD. IL-8 mRNA levels were quantified by RT-qPCR, and the relative expression of IL-8 mRNA was calculated in comparison to scrambled cells treated with glutamine and Q-VD. GCN2, ATF4, CHOP and TRAIL-R2 levels were assessed by western blotting. The data are presented as the mean ± SD from at least three independent experiments and were analysed via two-way ANOVA. ***P*<0.01; ****P<0.0001; ns = not statistically significant. Blots are representative of three independent experiments.

**Figure S2. Mechanisms underlying NF-κB activation and IL-8 induction in response to glutamine deprivation in colon cancer cell lines.** RKO cells were transfected with either a scrambled oligonucleotide (scr) or siRNAs targeting ATF4 **(A)** or p65 **(B)**. Forty-eight hours after transfection the cells were incubated for 24 hours in complete medium or glutamine-deprived medium, with 20 µM Q-VD. IL-8 mRNA levels were quantified by RT-qPCR, and the relative expression of IL-8 mRNA was calculated in comparison to scrambled cells treated with glutamine and Q-VD. ATF4 and p65 levels were assessed by western blotting. **C)** RKO cells were transfected with either a scrambled oligonucleotide (Scr) or a siRNA targeting caspase-8 (siC8) for 48 hours prior to treatment. To assess NF-κB transcriptional activity, cells were transfected with 200 ng of the pSI-Check2-RLuc-NF-κB-Firefly plasmid 24 hours before treatment. After 24 hours of glutamine deprivation with 20 µM Q-VD, luciferase activity was measured, and relative luciferase units (RLUs) were calculated in comparison to those of scrambled cells treated with glutamine and Q-VD. Caspase-8 knockdown in whole-cell extracts was confirmed by western blotting. **D)** RKO cells were transfected with either a scrambled oligonucleotide (scr) or a siRNA targeting caspase-8 (siC8). Forty-eight hours after transfection the cells were incubated for 24 hours in complete medium or glutamine-deprived medium, with 20 µM Q-VD. IL-8 mRNA levels were quantified by RT-qPCR, and the relative expression of IL-8 mRNA was calculated in comparison to scrambled cells treated with glutamine and Q-VD. **E)** Left panel, HCT116 cells were cultured in the presence or absence of 50 ng/mL TRAIL, and, where indicated, 250 ng/mL TRAIL-R2/DR5 Fc was added. Apoptosis was assessed after 24 hours of treatment. Right panel, HCT116 cells were cultured in the presence or absence of glutamine with 20 µM Q-VD for 16 hours. Where indicated, 250 ng/mL TRAIL-R2/DR5 Fc was added. IL-8 mRNA levels were assessed by RT-qPCR, and relative mRNA levels were determined in comparison to those in cells grown with glutamine and Q-VD (N.T.).

**Figure S3. Role of cFLIP in IL-8 induction and NF-kB activation upon glutamine deprivation. A) Left panel, RKO cells were** deprived of glutamine for the indicated time periods in the presence of Q-VD (20 µM). cFLIP_L_ and cFLIP_S_ levels were analyzed in whole-cell extracts by western blotting. Right panel, RKO pBABE-ø and pBABE-FLIPL cells were cultured with or without glutamine in the presence of 20 µM Q-VD for 24 hours. IL-8 mRNA levels were quantified by RT-qPCR. Relative IL-8 expression was calculated by comparing each condition to pBABE-ø cells maintained in glutamine and treated with Q-VD. cFLIP levels were assessed by western blotting. **B)** HCT116 cells were transfected with either a scrambled oligonucleotide (Scr) or a siRNA targeting cFLIP_S_ (siFS) for 48 hours prior to treatment. NF-κB transcriptional activity was assessed as described in Materials and Methods after 16 hours of glutamine deprivation with 20 µM Q-VD. Relative luciferase units (RLUs) were calculated in comparison to those of scrambled cells treated with glutamine and Q-VD. cFLIP_S_ knockdown in whole-cell extracts was confirmed by western blotting. **C)** HCT116 cells were transfected with either scrambled (Scr) or cFLIP_S_-targeting (siF_S_) oligonucleotide as described in B. Cells were either deprived or not deprived of glutamine in the presence of 20 µM Q-VD for 16 hours. IL-8 mRNA levels were assessed by RT-qPCR, and relative IL-8 mRNA levels were determined in comparison to those of scrambled cells incubated in the presence of glutamine and Q-VD. The data are presented as mean ± SD from three independent experiments and were analyzed by two-way ANOVA. ***P<0.001; ****P<0.0001.
