# Supplementary figures and images for: Activation of the integrated stress response and loss of cFLIPL under glutamine limitation induce IL-8 gene expression and secretion in glutamine-dependent tumor cells
Source: Cell Death Discov. 2025 Jul 19;11:332. doi: 10.1038/s41420-025-02625-3 (PMC12276259; doi:10.1038/s41420-025-02625-3)

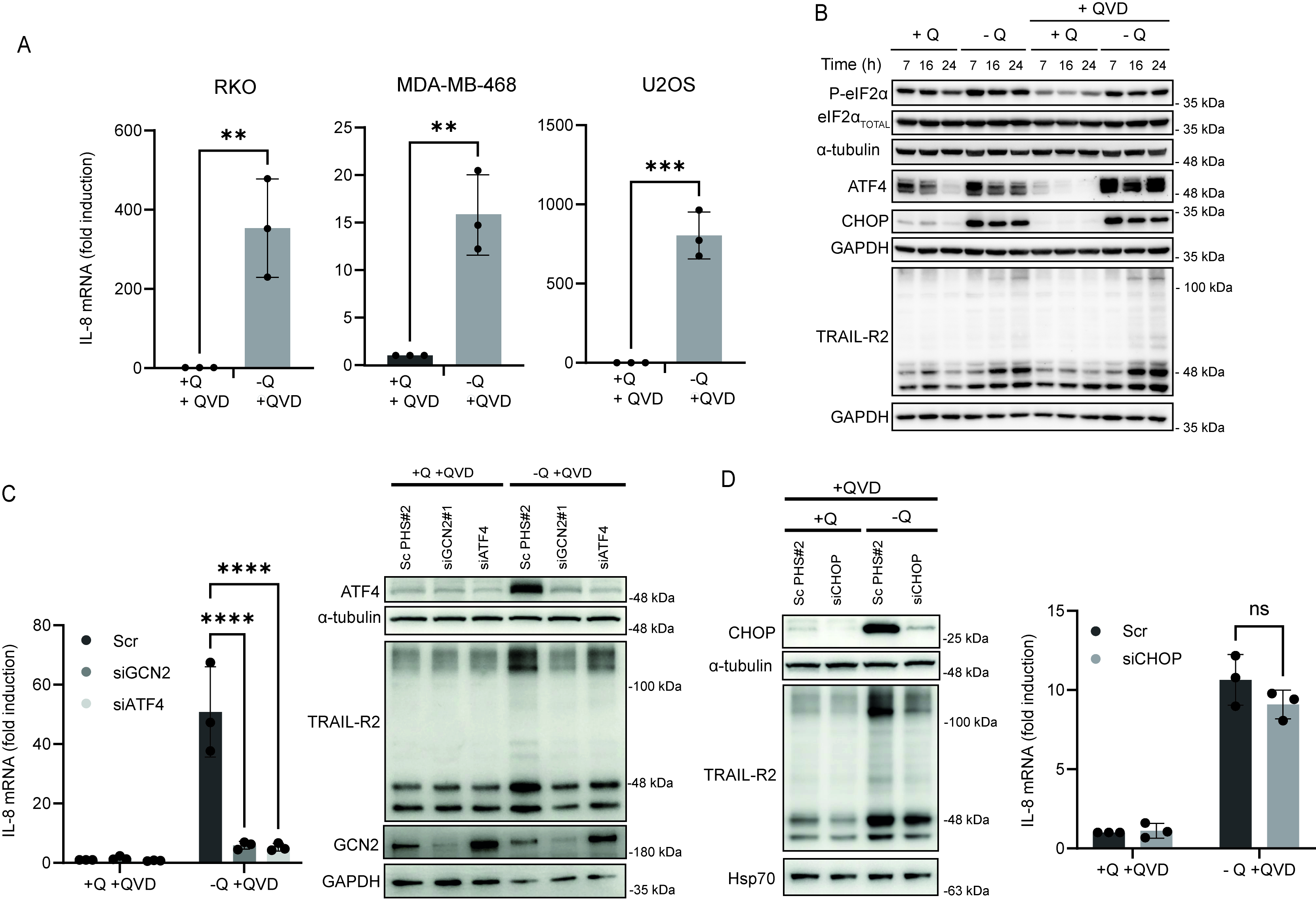

Supplement: Supplementary file 2 — Supplementary Figure 1 [file 41420_2025_2625_MOESM2_ESM.tif]

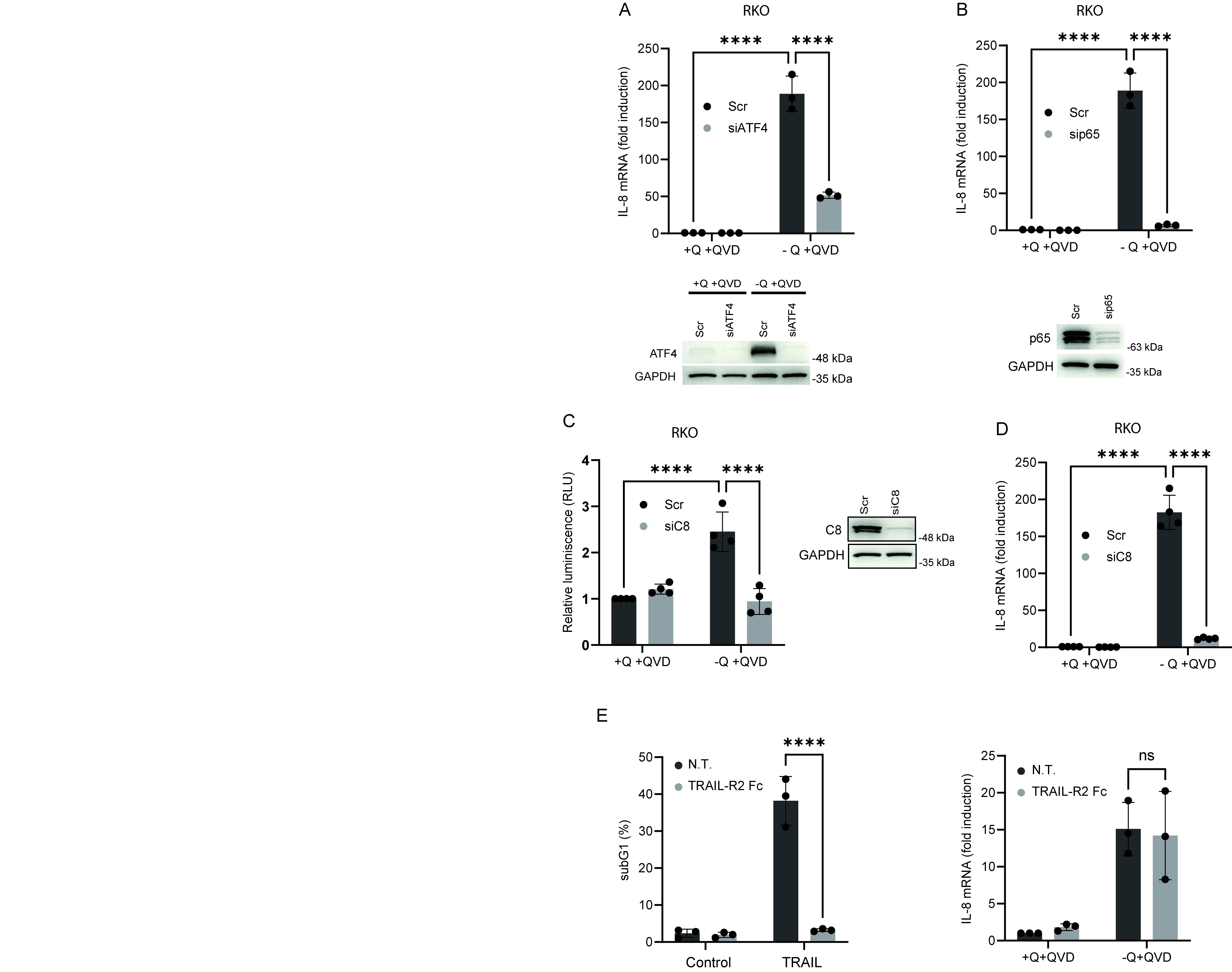

Supplement: Supplementary file 3 — Supplementary Figure 2 [file 41420_2025_2625_MOESM3_ESM.tif]

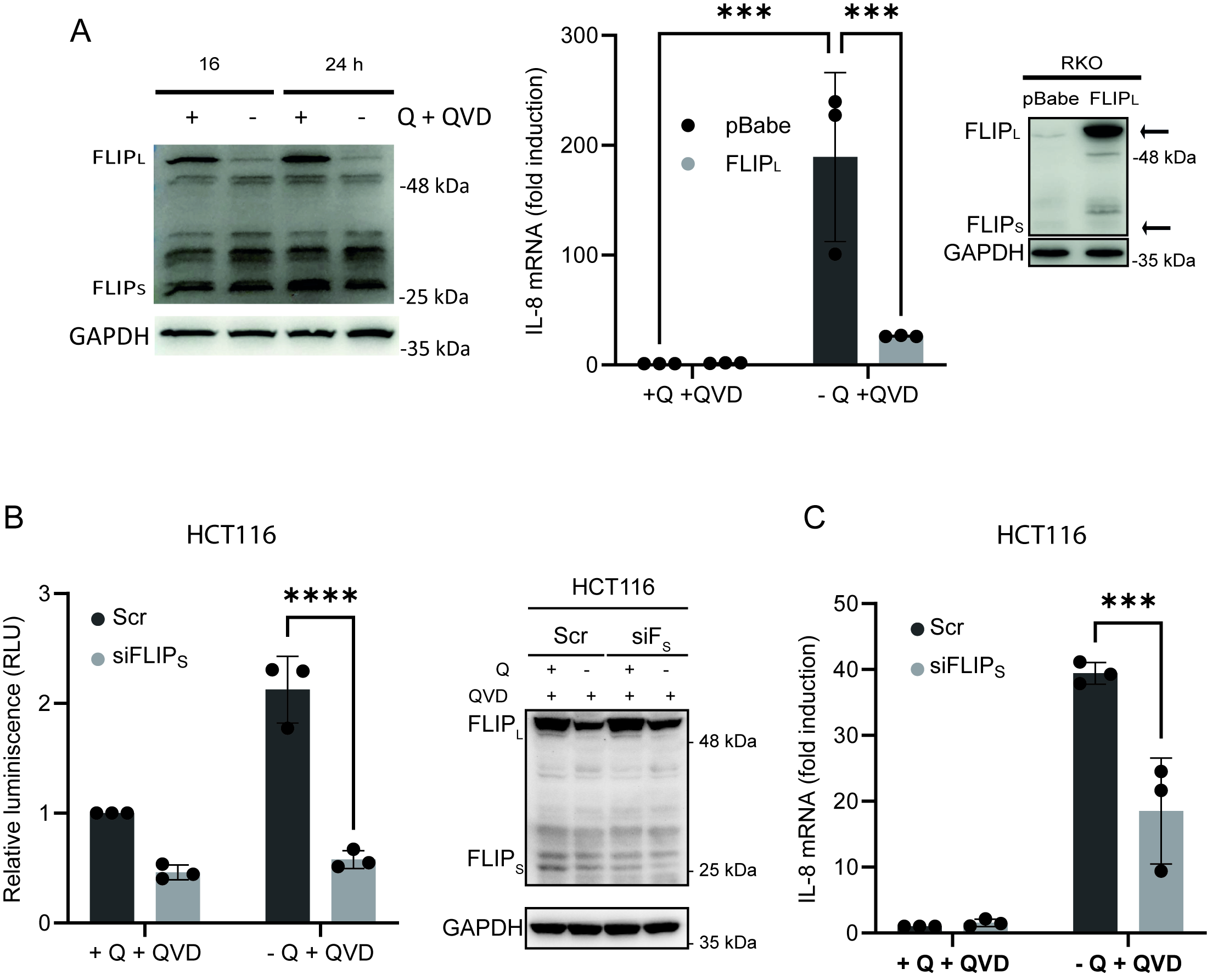

Supplement: Supplementary file 4 — Supplementary Figure 3 [file 41420_2025_2625_MOESM4_ESM.tif]
