# Supplementary material for: Activation of the integrated stress response and loss of cFLIPL under glutamine limitation induce IL-8 gene expression and secretion in glutamine-dependent tumor cells
Source: Cell Death Discov. 2025 Jul 19;11:332. doi: 10.1038/s41420-025-02625-3 (PMC12276259; doi:10.1038/s41420-025-02625-3)
Supplement: Supplementary file 5 — Original data [file 41420_2025_2625_MOESM5_ESM.pdf]

Fig. 1E

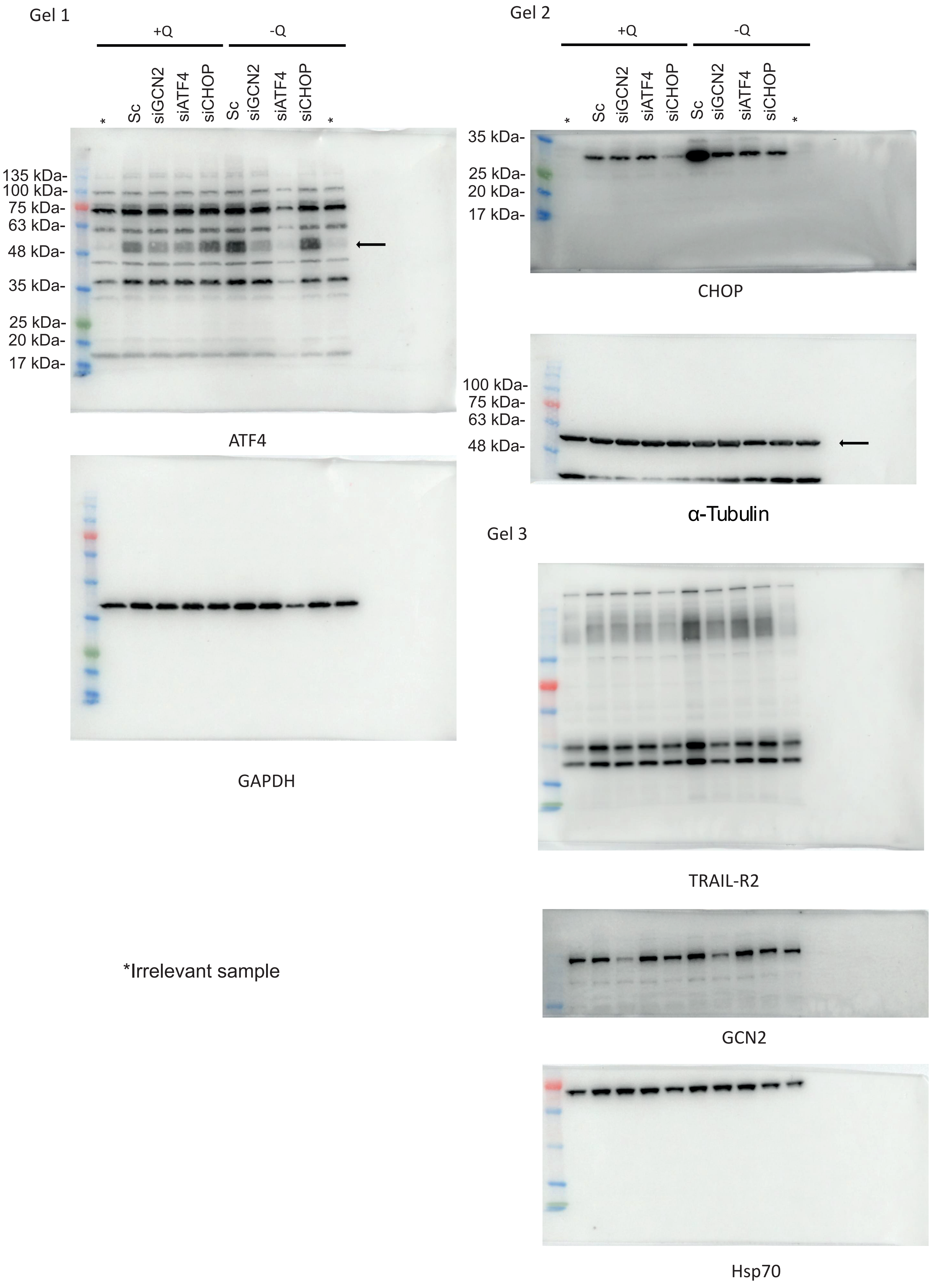

Gel 1
+Q
-Q
*
Sc
siGCN2
siATF4
siCHOP
Sc
siGCN2
siATF4
siCHOP
*
135 kDa-
100 kDa-
75 kDa-
63 kDa-
48 kDa-
35 kDa-
25 kDa-
20 kDa-
17 kDa-
ATF4
GAPDH
*Irrelevant sample
Gel 2
+Q
-Q
*
Sc
siGCN2
siATF4
siCHOP
Sc
siGCN2
siATF4
siCHOP
*
35 kDa-
25 kDa-
20 kDa-
17 kDa-
CHOP
100 kDa-
75 kDa-
63 kDa-
48 kDa-
α-Tubulin
Gel 3
TRAIL-R2
GCN2
Hsp70

Fig. 2A_B

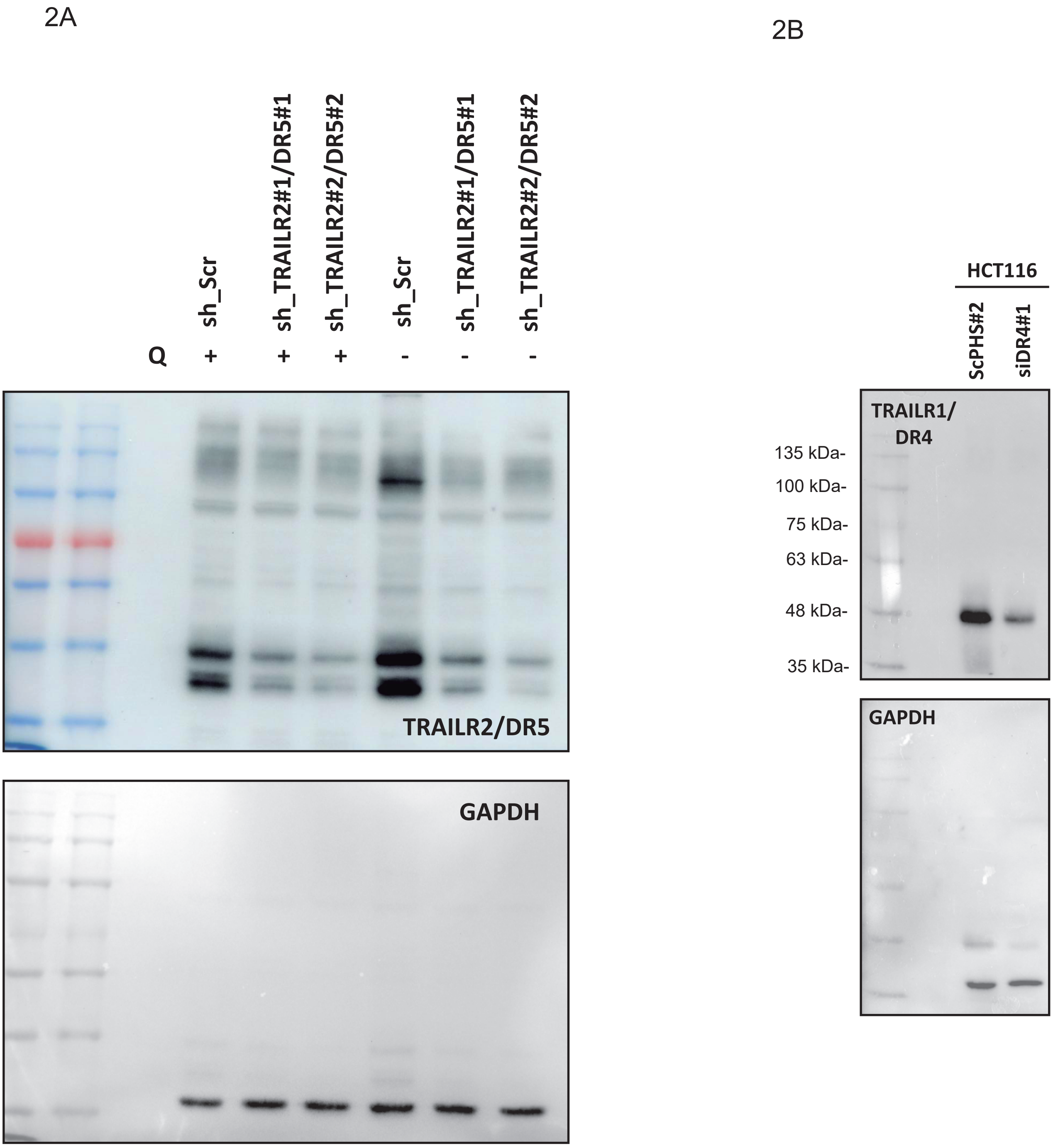
2A
2B
Q
+
sh_Scr
+
sh_TRAILR2#1/DR5#1
+
sh_TRAILR2#2/DR5#2
-
sh_Scr
-
sh_TRAILR2#1/DR5#1
-
sh_TRAILR2#2/DR5#2
TRAILR2/DR5
GAPDH
HCT116
ScPHS#2
siDR4#1
TRAILR1/
DR4
135 kDa-
100 kDa-
75 kDa-
63 kDa-
48 kDa-
35 kDa-
GAPDH

Fig. 3B_C

3B

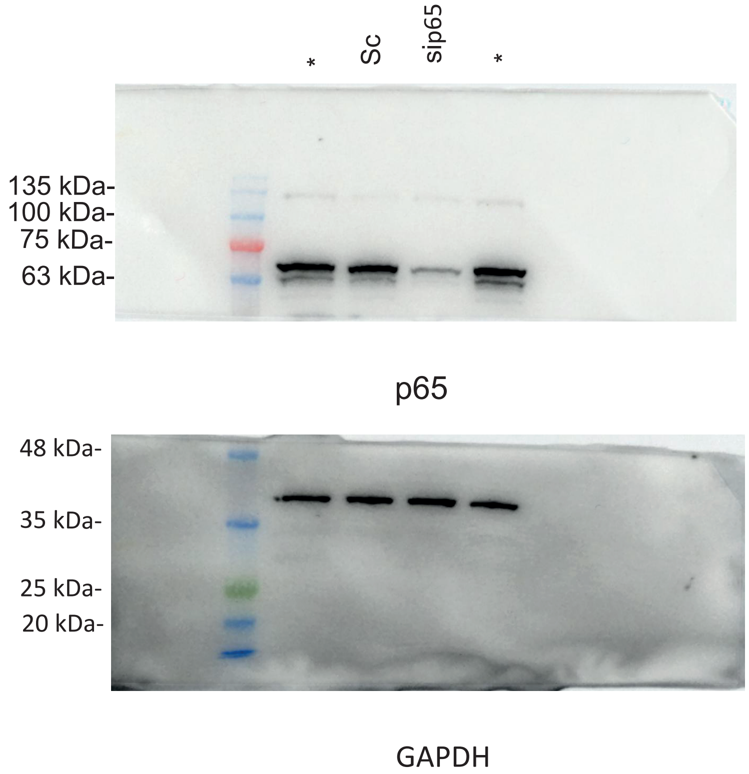

*Irrelevant sample

3C

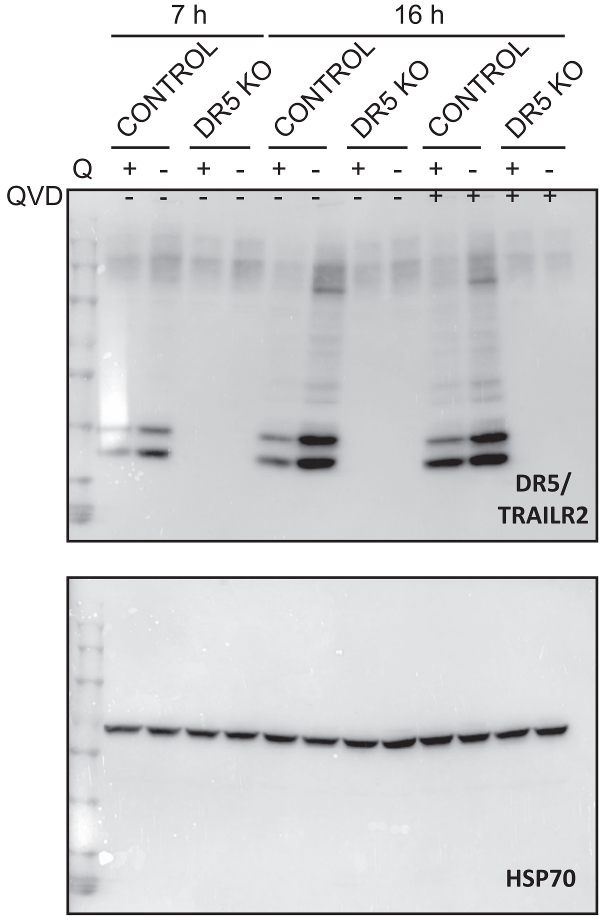

3E Gel 1

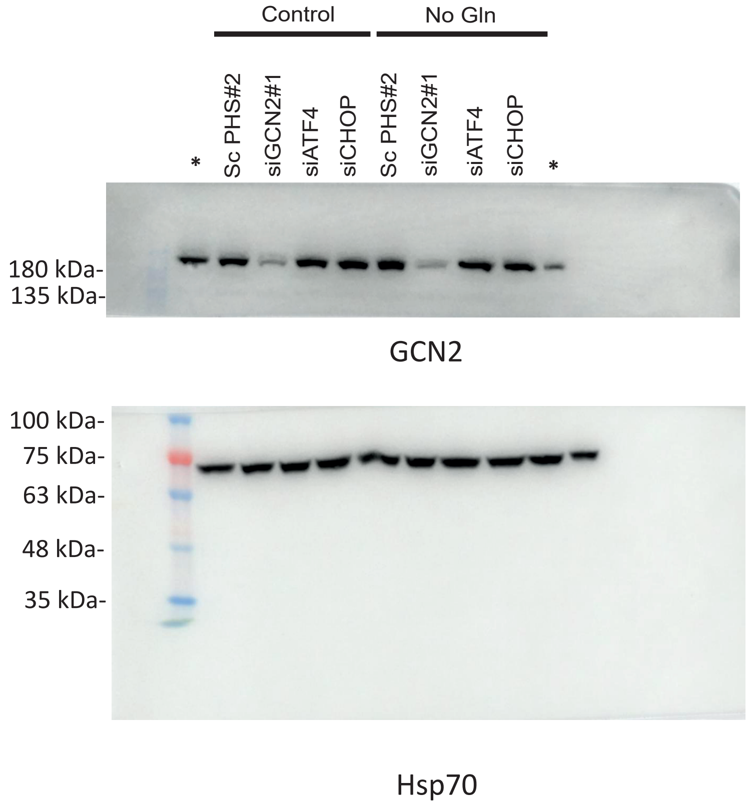

3E Gel 2

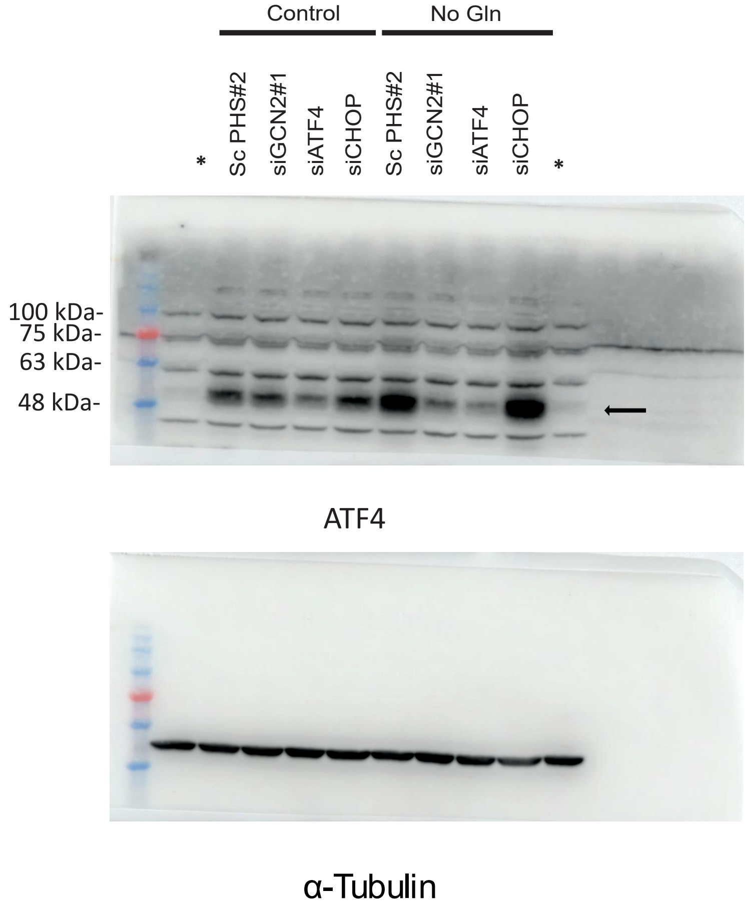

*Irrelevant sample

Fig. 4

4A

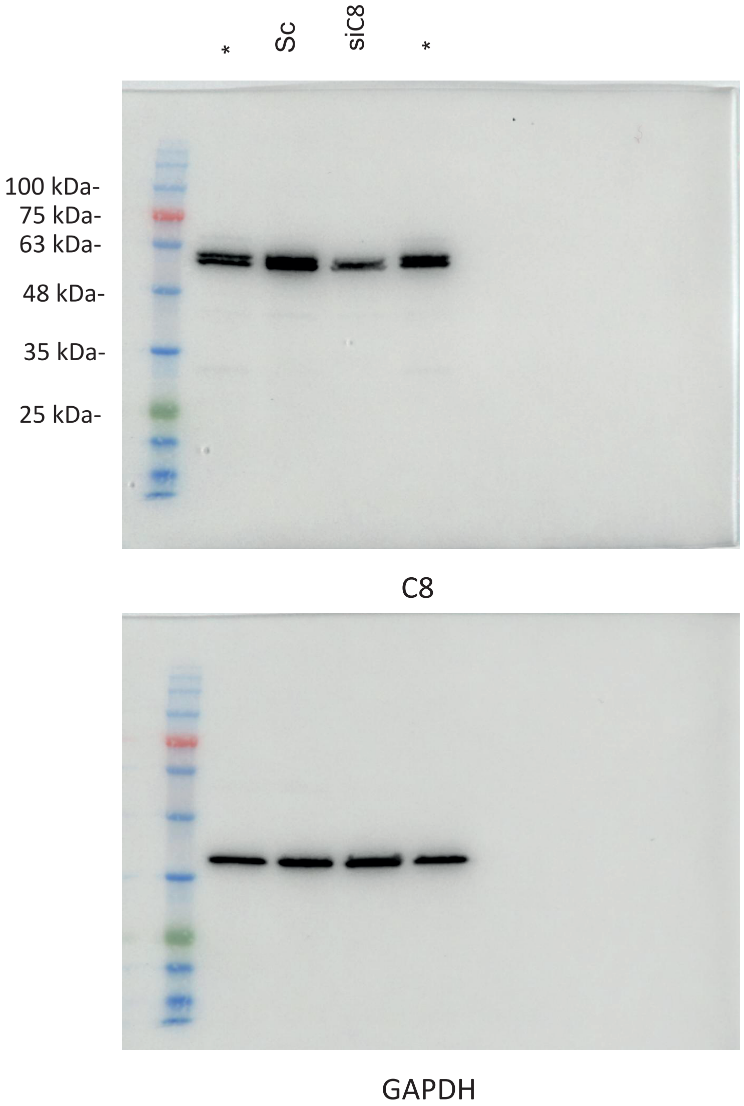

4C

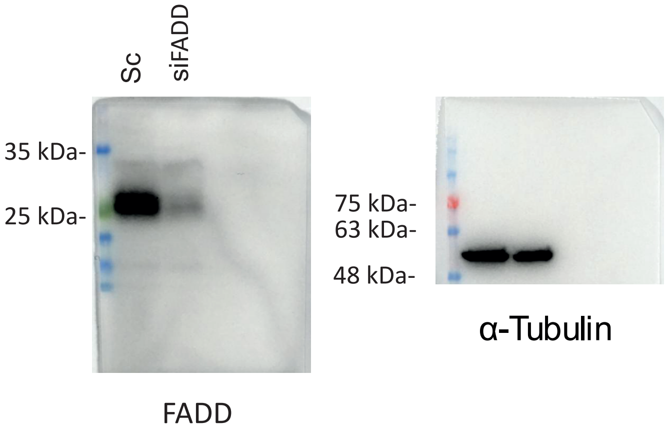

4E

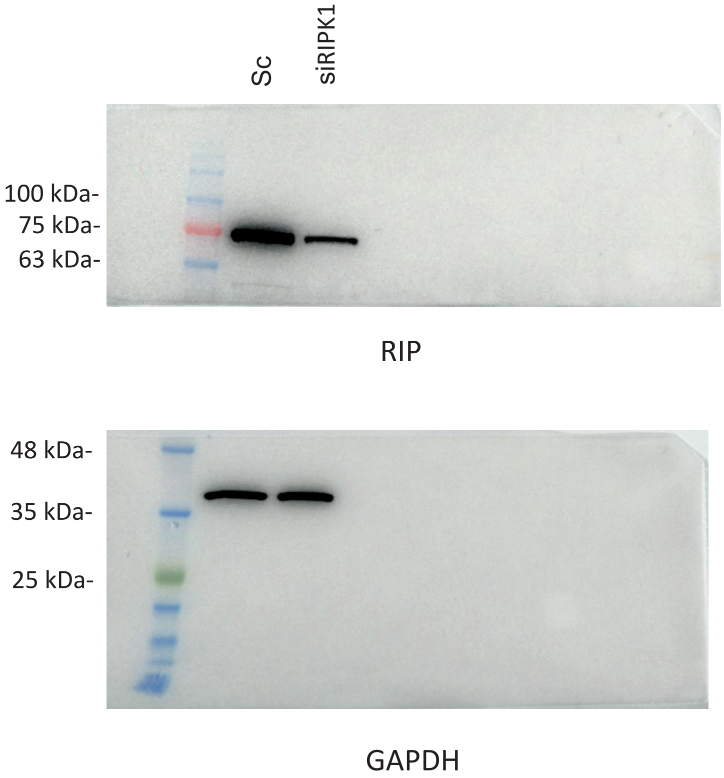

4G

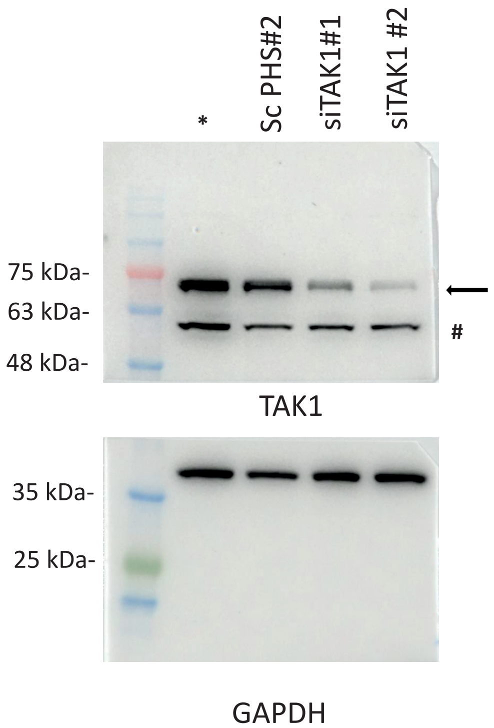

*Irrelevant sample

#Unespecific band

Fig. 5

5A

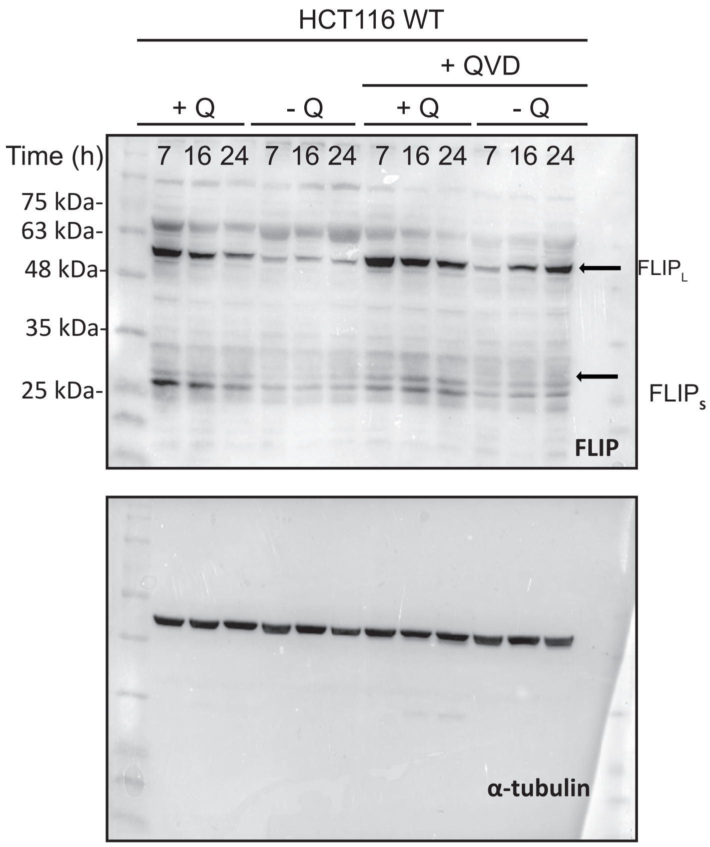

5B

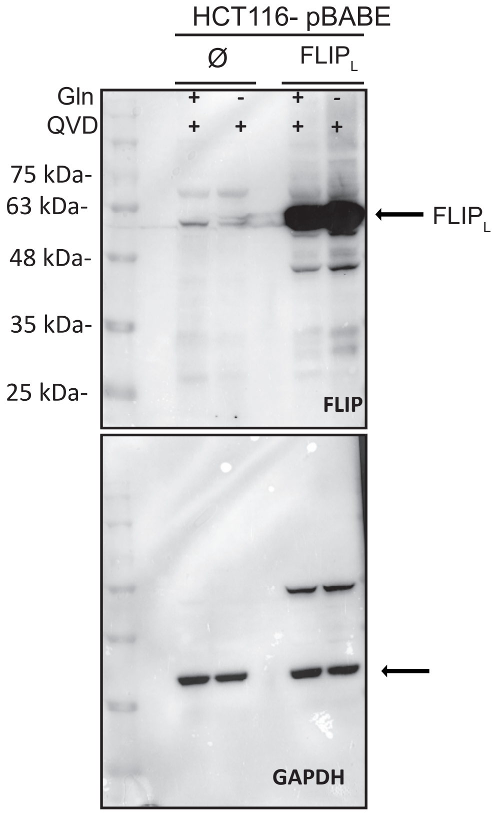

5D

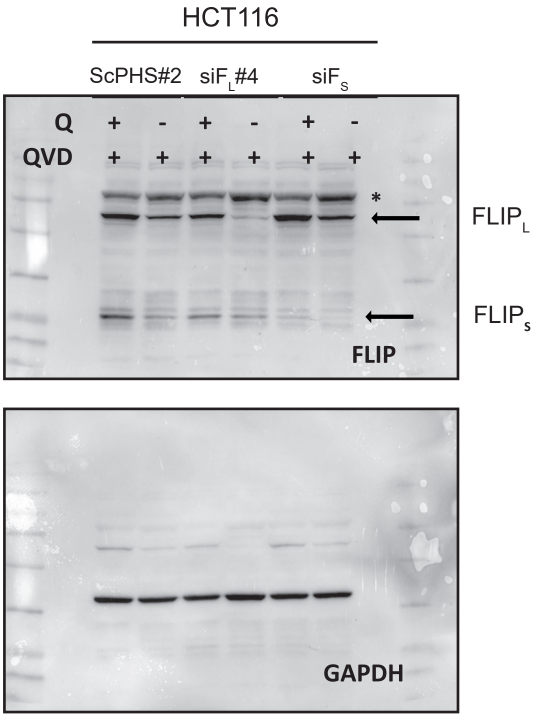

*Unespecific band

Fig. 6

6C

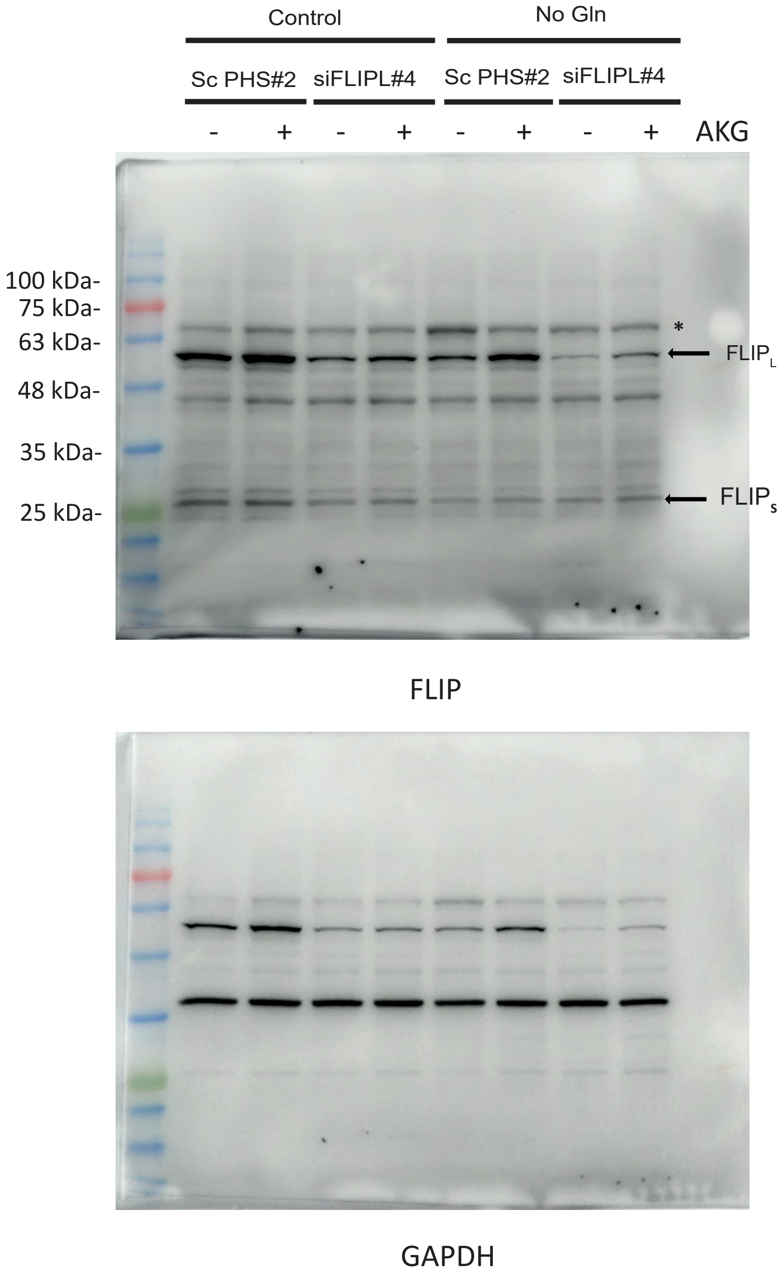

*Unespecific band

Fig. S1B

Gel 1: It's the same as in Figure 5A;
first, we look at FLIP, and then p-eIF2a,
eIf2a and Tubulin

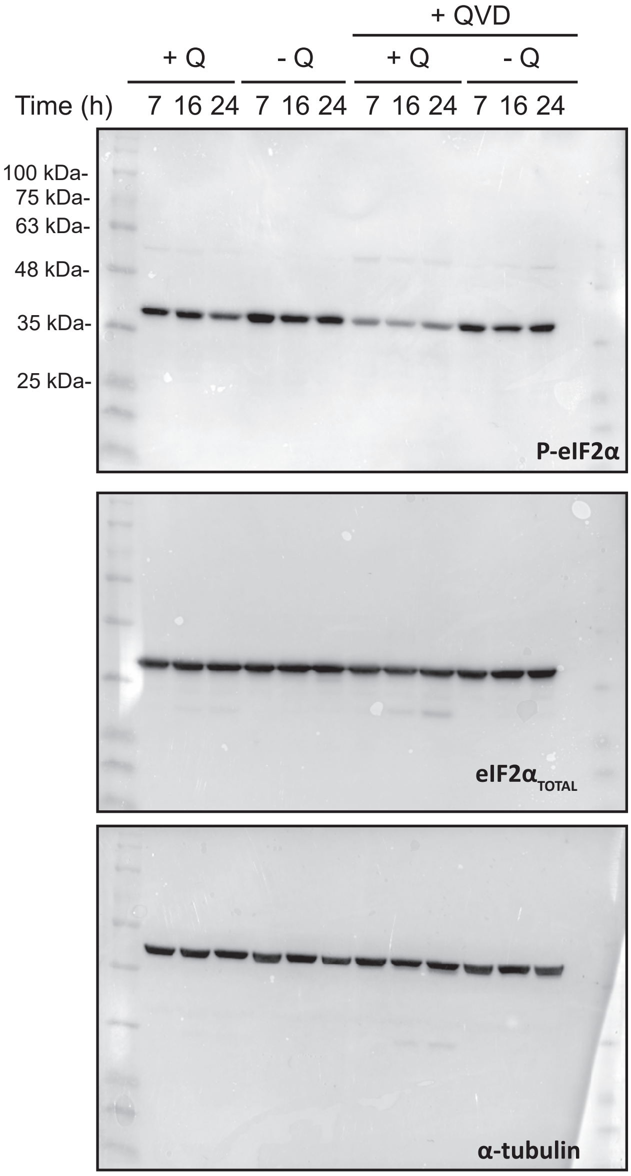

Gel 2

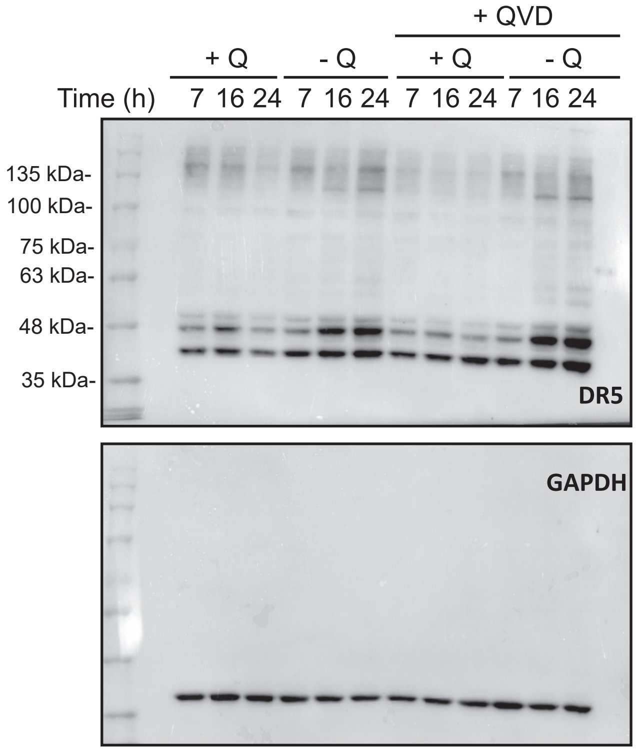

Gel 3

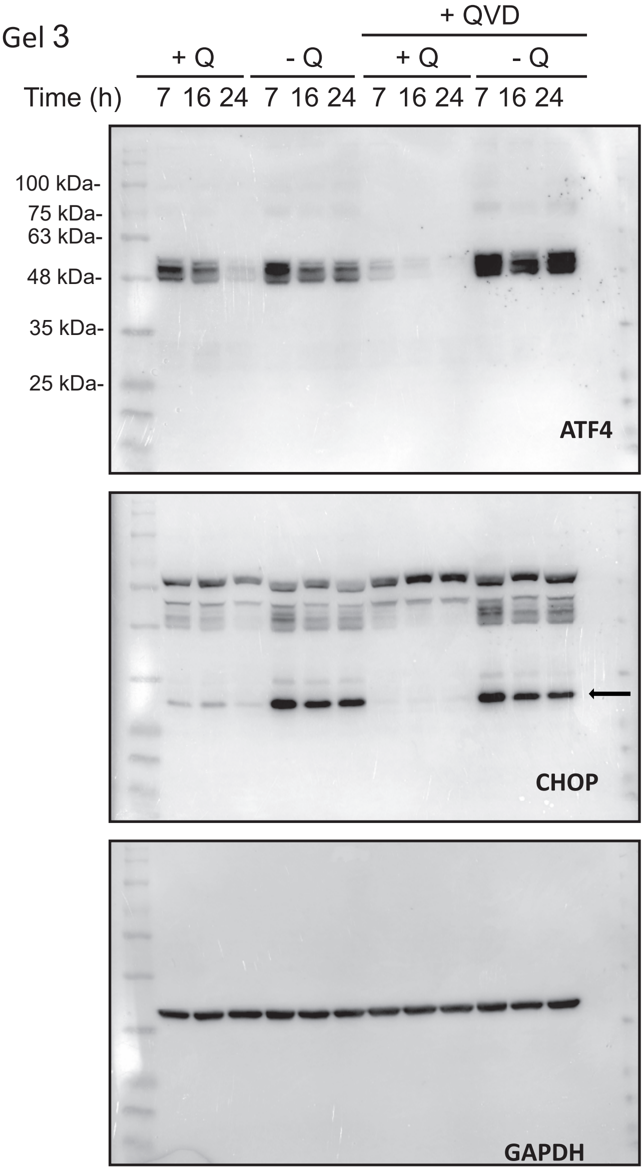

Fig. S1C

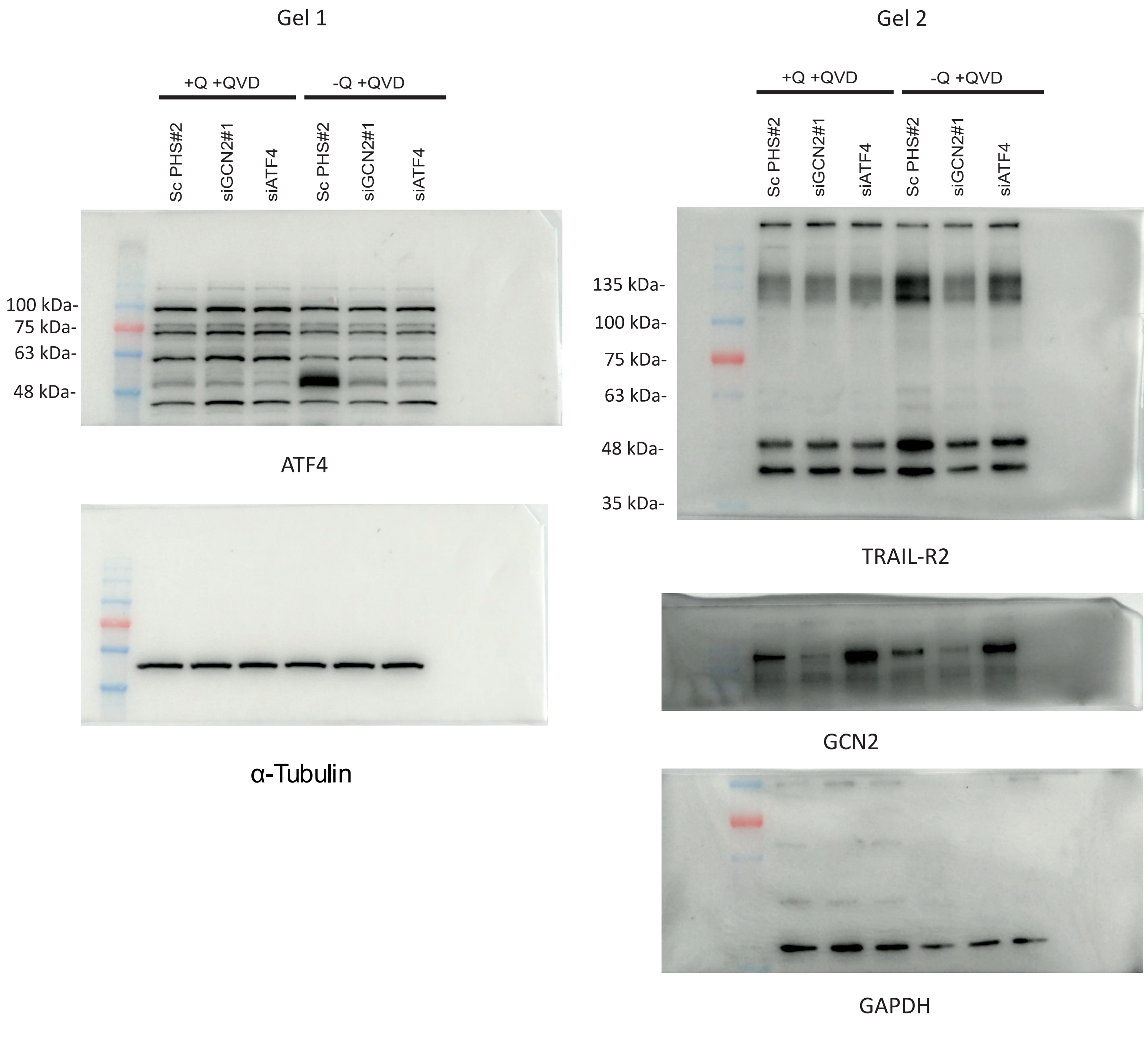
Gel 1
+Q +QVD
-Q +QVD
Sc PHS#2
siGCN2#1
siATF4
Sc PHS#2
siGCN2#1
siATF4
100 kDa-
75 kDa-
63 kDa-
48 kDa-
ATF4
α-Tubulin
Gel 2
+Q +QVD
-Q +QVD
Sc PHS#2
siGCN2#1
siATF4
Sc PHS#2
siGCN2#1
siATF4
135 kDa-
100 kDa-
75 kDa-
63 kDa-
48 kDa-
35 kDa-
TRAIL-R2
GCN2
GAPDH

Fig. S1D

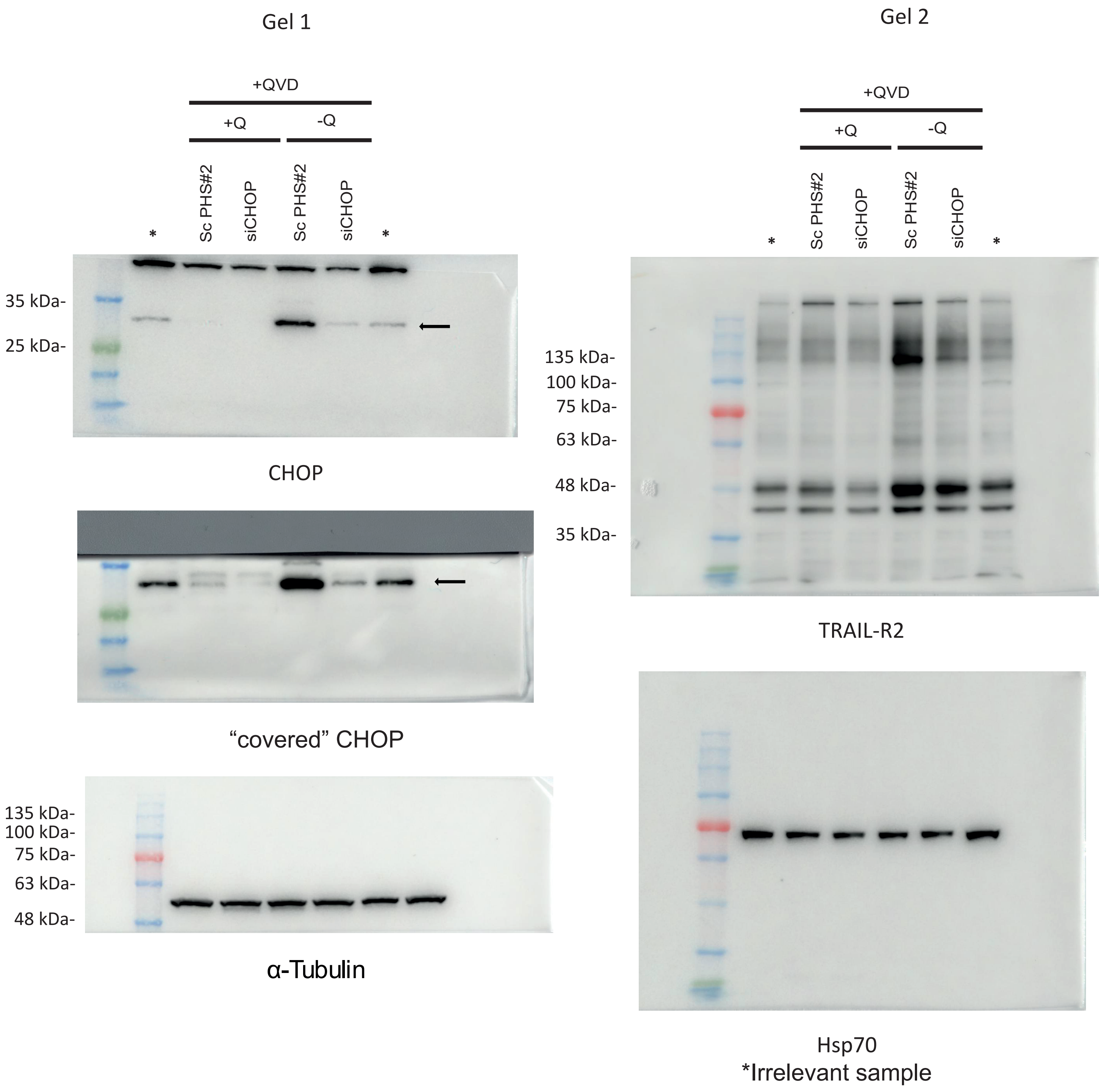

Gel 1
+QVD
+Q
-Q
*
Sc PHS#2
siCHOP
Sc PHS#2
siCHOP
*
35 kDa-
25 kDa-
CHOP
“covered” CHOP
135 kDa-
100 kDa-
75 kDa-
63 kDa-
48 kDa-
α-Tubulin
Gel 2
+QVD
+Q
-Q
*
Sc PHS#2
siCHOP
Sc PHS#2
siCHOP
*
135 kDa-
100 kDa-
75 kDa-
63 kDa-
48 kDa-
35 kDa-
TRAIL-R2
Hsp70
*Irrelevant sample

Fig. S2 A_B_C

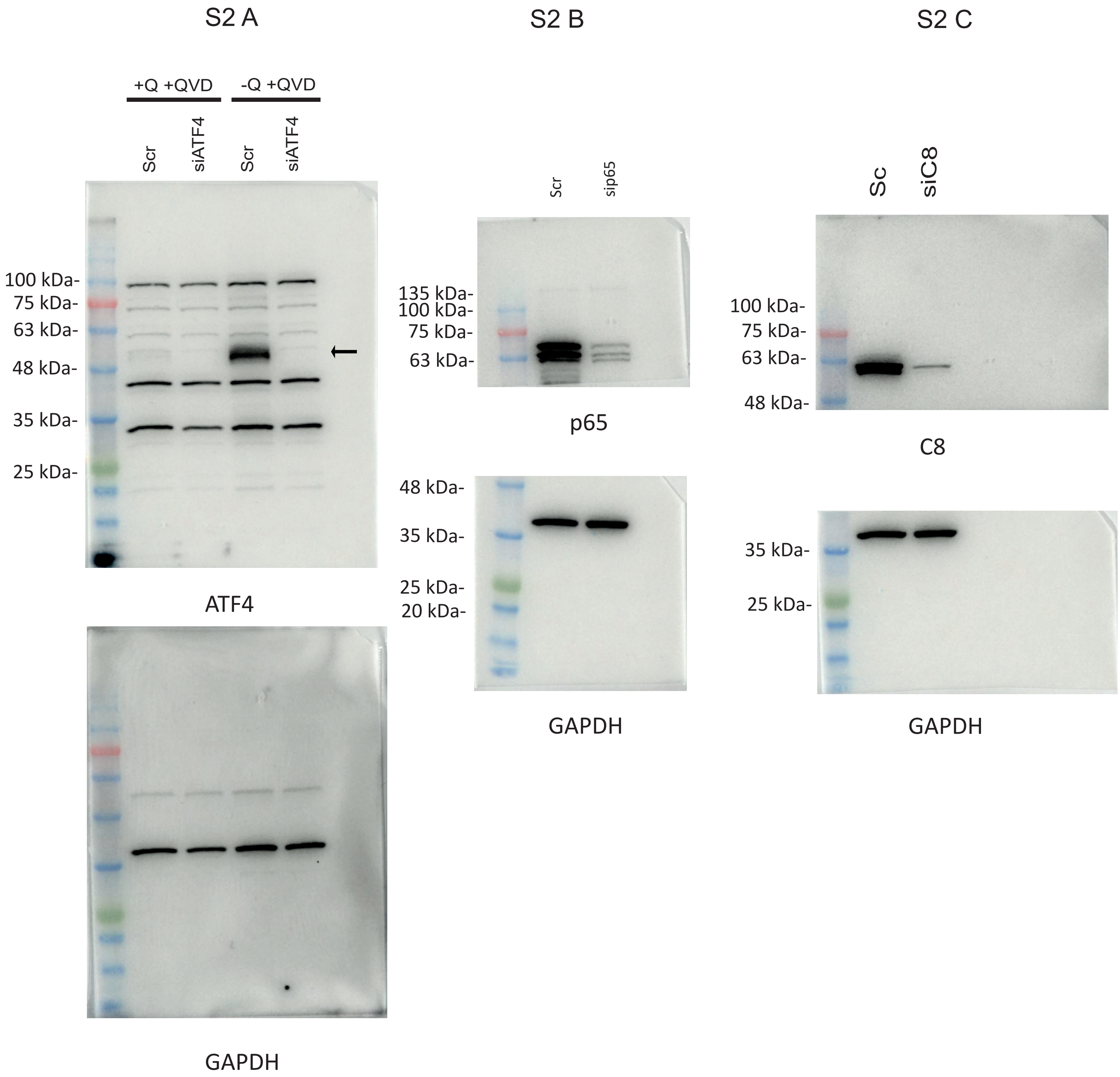
S2 A
+Q +QVD
-Q +QVD
Scr
siATF4
Scr
siATF4
100 kDa-
75 kDa-
63 kDa-
48 kDa-
35 kDa-
25 kDa-
ATF4
GAPDH
S2 B
Scr
sip65
135 kDa-
100 kDa-
75 kDa-
63 kDa-
p65
48 kDa-
35 kDa-
25 kDa-
20 kDa-
GAPDH
S2 C
Sc
siC8
100 kDa-
75 kDa-
63 kDa-
48 kDa-
C8
35 kDa-
25 kDa-
GAPDH

Fig. S3

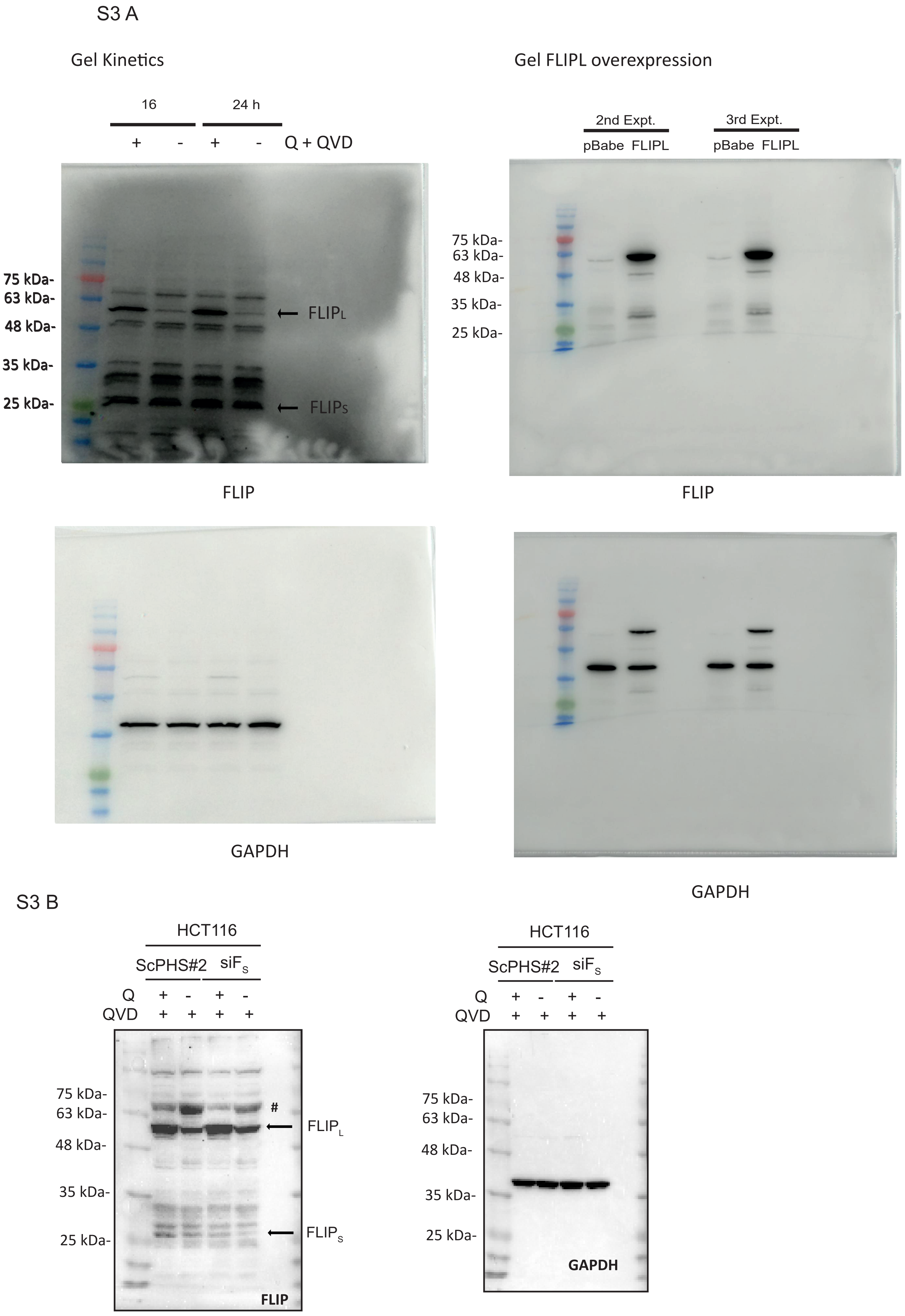
S3 A
Gel Kinetics
16
24 h
+ - + - Q + QVD
75 kDa-
63 kDa-
48 kDa-
35 kDa-
25 kDa-
FLIPL
FLIPS
FLIP
GAPDH
Gel FLIPL overexpression
2nd Expt.
3rd Expt.
pBabe FLIPL
pBabe FLIPL
75 kDa-
63 kDa-
48 kDa-
35 kDa-
25 kDa-
FLIP
GAPDH
S3 B
HCT116
ScPHS#2
siFS
Q + - + -
QVD + + + +
75 kDa-
63 kDa-
48 kDa-
35 kDa-
25 kDa-
#
FLIPL
FLIPS
FLIP
HCT116
ScPHS#2
siFS
Q + - + -
QVD + + + +
75 kDa-
63 kDa-
48 kDa-
35 kDa-
25 kDa-
GAPDH
